# The identification of effective tumor-suppressing neoantigens using a tumor-reactive TIL TCR-pMHC ternary complex

**DOI:** 10.1038/s12276-024-01259-2

**Published:** 2024-06-12

**Authors:** Sang Hoon Kim, Bo Ryeong Lee, Sung-Min Kim, Sungsik Kim, Min-seok Kim, Jaehyun Kim, Inkyu Lee, Hee-Soo Kim, Gi-Hoon Nam, In-San Kim, Kyuyoung Song, Yoonjoo Choi, Dong-Sup Lee, Woong-Yang Park

**Affiliations:** 1 Geninus Inc., Seoul, 05836 Korea; 2 Department of Research and Development, SHIFTBIO Inc., Seoul, 02751 Korea; 3 KU-KIST Graduate School of Converging Science and Technology, Korea University, Seoul, 02841 Korea; 4 Department of Biochemistry and Molecular Biology, Korea University College of Medicine, Seoul, 02841 Korea; 5 Chemical & Biological Integrative Research Center, Biomedical Research Division, Korea Institute of Science and Technology (KIST), Seoul, 02792 Korea; 6 Combinatorial Tumor Immunotherapy MRC, Chonnam National University Medical School, Hwasun-gun, Jeollanam-do 58128 Korea; 7 Department of Biomedical Sciences, Seoul National University College of Medicine, Seoul, Korea; 8 Department of Health Science and Technology, Samsung Advanced Institute of Health Science and Technology, Sungkyunkwan University, Seoul, Korea; 9 Samsung Genome Institute, Samsung Medical Center, Sungkyunkwan University School of Medicine, Seoul, Korea

**Keywords:** Cancer immunotherapy, Peptide vaccines, Machine learning

## Abstract

Neoantigens are ideal targets for cancer immunotherapy because they are expressed de novo in tumor tissue but not in healthy tissue and are therefore recognized as foreign by the immune system. Advances in next-generation sequencing and bioinformatics technologies have enabled the quick identification and prediction of tumor-specific neoantigens; however, only a small fraction of predicted neoantigens are immunogenic. To improve the predictability of immunogenic neoantigens, we developed the in silico neoantigen prediction workflows VACINUS_pMHC_ and VACINUS_TCR:_ VACINUS_pMHC_ incorporates physical binding between peptides and MHCs (pMHCs), and VACINUS_TCR_ integrates T cell reactivity to the pMHC complex through deep learning-based pairing with T cell receptors (TCRs) of putative tumor-reactive CD8 tumor-infiltrating lymphocytes (TILs). We then validated our neoantigen prediction workflows both in vitro and in vivo in patients with hepatocellular carcinoma (HCC) and in a B16F10 mouse melanoma model. The predictive abilities of VACINUS_pMHC_ and VACINUS_TCR_ were confirmed in a validation cohort of 8 patients with HCC. Of a total of 118 neoantigen candidates predicted by VACINUS_pMHC_, 48 peptides were ultimately selected using VACINUS_TCR_. In vitro validation revealed that among the 48 predicted neoantigen candidates, 13 peptides were immunogenic. Assessment of the antitumor efficacy of the candidate neoepitopes using a VACINUS_TCR_ in vivo mouse model suggested that vaccination with the predicted neoepitopes induced neoantigen-specific T cell responses and enabled the trafficking of neoantigen-specific CD8 + T cell clones into the tumor tissue, leading to tumor suppression. This study showed that the prediction of immunogenic neoantigens can be improved by integrating a tumor-reactive TIL TCR-pMHC ternary complex.

## TCR-Paired Neoantigen Prediction Enhances T Cell Response for Cancer Therapy

Cancer scientists have created a new way to identify and choose neoantigens (new proteins on cancer cells) for use in cancer vaccines. The research, led by Woong-Yang Park, included 33 patients with different cancer types. The scientists used a mix of computer-based prediction techniques and lab-based tests to find the most effective neoantigens. The results showed that the new technique, named VACINUS, was better at predicting effective neoantigens than older methods. The scientists concluded that the VACINUS technique could improve the immune response of predicted neoantigens, making them better at activating an immune reaction against cancer cells. This could result in the creation of more effective cancer vaccines in the future.

This summary was initially drafted using artificial intelligence, then revised and fact-checked by the author.

## Introduction

Neoantigens have been characterized as promising therapeutic targets for cancer immunotherapy because they are newly expressed peripherally on cancer tissue and, thus, are not subject to thymic central tolerance^1^. During the progression of cancer, the tumor immune microenvironment becomes immunosuppressive, which compromises the activation and effector capacity of cancer-specific T cells. In these circumstances, vaccines designed to target tumor-specific neoepitopes are promising for effective tumor control.

Vaccines targeting mutant proteins present in tumor cells have been successful in mouse models and clinical studies. In humans, neoantigen vaccine studies have shown the ability to generate neoantigen-specific T cells in melanoma, glioblastoma, and pancreatic cancer^2–4^ and are supportive of the ability of these therapies to induce vaccine-related tumor regression in melanoma^2^ and to protect against tumor recurrence in melanoma and pancreatic cancer^4,5^. Neoantigen-targeting therapies are thus viewed as effective approaches for generating antitumor immune responses^6,7^, and clinical trials have revealed that personalized neoantigen-based cancer vaccines are feasible, safe, and effective in patients with melanoma, glioblastoma, breast cancer, and pancreatic cancer^8^.

The efficacy of cancer vaccines primarily depends on the selection of highly immunogenic neoantigens that can induce the strong cytotoxic CD8+ T cell response required to reject tumors. Foreign antigens or highly expressed tumor-specific aberrant proteins are proteolytically cleaved by the proteasome, and the resulting mutated peptides are presented on the membrane of malignant cells via the peptide-MHC class I complex (pMHC). The activation of CD8+ T cells is fundamentally dependent on the precise interaction between T cell receptors (TCRs) and exogenous antigenic peptides presented via MHC class I. Thus, the interaction between the pMHC complex and TCR is of paramount importance.

We developed in silico neoantigen prediction workflows, VACINUS_pMHC_ and VACINUS_TCR_, to predict tumor neoantigens more accurately from raw sequencing data based on peptide-MHC binding and a putative tumor-reactive tumor-infiltrating lymphocyte (TIL) TCR-pMHC ternary complex, respectively. Using a B16F10 mouse tumor model, we showed that these workflows can be used to better select therapeutically relevant cancer vaccines.

## Materials and methods

### Patients

This study was approved by the institutional review board (IRB) of Asan Medical Center (IRB number: 2022-0263), and informed consent was obtained from all participants. A total of 33 patients were recruited: 6 with colorectal cancer (CRC), 2 with melanoma, 14 with hepatocellular carcinoma (HCC), and 11 with gastric cancer (GC). Of the 33 patients, 8 patients with HCC were assigned to the validation cohort for neoantigen prediction workflows, while the remaining 25 patients composed the training cohort. All procedures were carried out in accordance with relevant guidelines and regulations.

### Identification of potential neoantigens using next-generation sequencing (NGS) data

The neoantigen selection process was based on the following features: NGS data, peptide/MHC-I interactions, and TCR-pMHC interactions. First, we selected highly expressed nonsynonymous or indel mutations using whole transcriptome sequencing (WTS) data. The selection criteria included VAF > 0, read counts > 10 at the transcript level, and the sum of transcripts per million (TPM) calculated based on genes > log 0.4. Second, an in silico prediction method was employed to assess the binding affinity of the mutated peptides to the HLA class I molecules of the patient. It is known that there is a correlation between immunogenicity and high-affinity binding to HLA class I molecules^9^, and in general, in silico prediction tools have high positive predictive value in the identification of strong binders^10^. Only peptides with 9–10 amino acids were considered. We used MHCflurry (v2.0.1)^11^ to estimate the binding affinity of the pMHC-I complex. In the selection process, the second amino acid is an important binding anchor position; thus, any mutations in the second position were excluded. Third, the binding characteristics of the peptides presented on the HLA molecules to the TCR were considered. The interaction between the TCR and the pMHC complex has been shown to impact immunogenicity^12,13^. We applied PRIME, which combines predictions of binding to MHC-I molecules and propensity for TCR recognition^14^, and obtained a PRIME score corresponding to alleles based on immunogenicity. Finally, we applied an ensemble model built using various features, including immune-related parameters such as peptide-MHC affinity, dissimilarity, and foreignness (see the section “Results”).

### Prioritization of immunogenic neoantigens

Using scRNA-seq data of CD8+ T cells isolated from TILs, we performed a standard clustering process using the Louvain method to identify putative tumor-reactive CD8+ T cells. Previous studies reported exhausted T cells and proliferative T cells as tumor-reactive CD8+ T cells^15–17^. To maximize the number of putative tumor-reactive T cells present in the tumor tissue, we additionally included activated and effector T cells. CD8+ T cell subtype markers were obtained mainly from the report by Oliveira et al.; proliferating T cells (*MKI67, TOP2A*), exhausted T cells (*PDCD1, TIGIT, HAVCR2, LAG3, CTLA4*), effector T cells (*GZMA, GZMK, GZMH, PRF1, NKG7, TNF, GNLY, IFNG*), and activated T cells (*HLA-DR*)^15–18^. The remaining T cell types were classified as naive/memory T cells (*SELL, CCR7, and IL7R*), NK/γδ-like T cells (*TRGV9, TRDV2, KLRB1, and KLRC3*), and MAIT cells (*TRAV1-2*)^19^.

For prioritizing the immunogenic tumor neoantigen candidates, peptides were binary-classified as “Tier 1” (high priority) or “Nontier 1” based on the binding between the TCR of the putative tumor-reactive TILs and pMHC complexes. The binding specificities between TCRs and peptide sequences presented on the HLA molecules were predicted using pMTnet (v1.0)^20^. While the default cutoff value for pMTnet is 0.01, we opted for a more inclusive approach by setting the threshold higher, using 0.05 as the cutoff to expand our pool of candidate peptides.

## Results

### Prefiltration criteria for the selection of candidate neoantigens

**Fig. 1:**
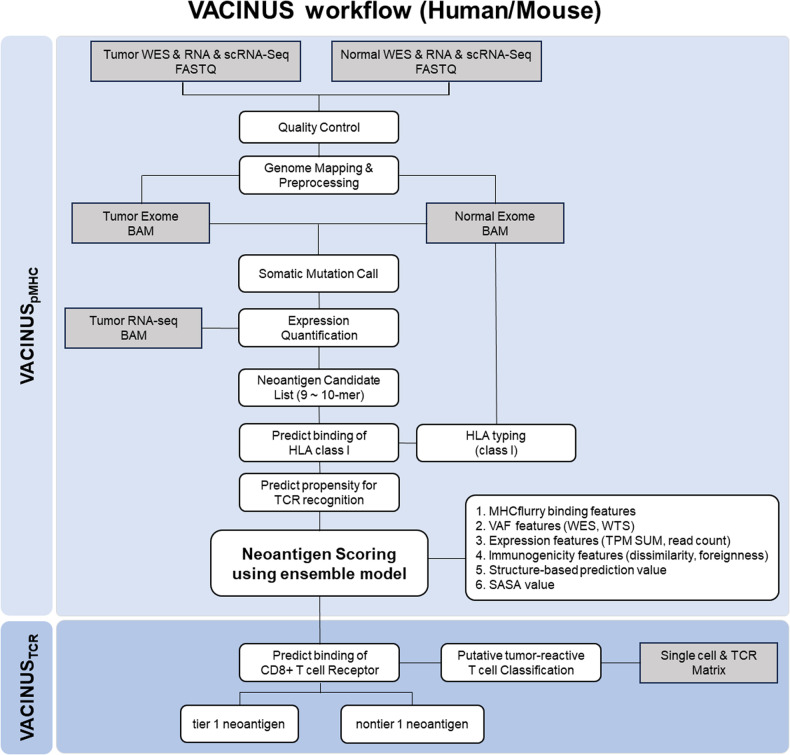
VACINUS workflow for neoantigen prediction and tier selection.

The workflow of neoantigen identification is shown in Fig. 1, which integrates commonly used bioinformatics tools (Supplementary Table 1). To establish prefiltration criteria for the selection of candidate neoantigens, we used our in-house immunogenicity dataset and the Tumor Neoantigen Selection Alliance (TESLA) dataset^21^. To determine the prefiltering criteria, we used only the results for immunogenic neoantigens from both datasets. Three patients with melanoma (1, 2, 3) and 2 patients with non-small cell lung cancer (NSCLC) (12, 16) in the TESLA dataset provided 25 immunogenic neoantigens. The in-house data included the results of in vitro immunogenicity screening of neoantigens from a training cohort of 25 patients (CRC: 6, GC: 11, HCC: 6, MN: 2). The neoantigens comprised 27 immunogenic neoantigens (data not shown). Only 9- and 10-mer peptides and a WTS VAF > 0 were considered. Taking the second anchoring position into account, any peptides that contained mutations at the second position were excluded. We tried to maintain high sensitivity in the selection process of potential neoantigen candidates and thus set cutoff values accordingly. We used log 0.4 as the TPM cutoff for both datasets (Supplementary Fig. 1a). For MHCflurry and PRIME, 140 nM and 0.847 (PRIME %rank score) were used since the cutoff values maintained 90% sensitivity in both datasets (Supplementary Fig. 1b, c). Applying these cutoff criteria, 2 immunogenic neoantigens were filtered out from the in-house dataset, resulting in a total of 25 immunogenic neoantigens available for VACINUS_pMHC_ modeling.

### Development of an in silico neoantigen prediction workflow, VACINUS_pMHC_

The TESLA dataset produced 199 neoantigens that passed the prefiltration criteria for selecting candidate neoantigens, of which 25 were positive for immunogenicity. In the in-house dataset, there were 99 neoantigens consisting of 25 immunogenicity-positive and 74 immunogenicity-negative neoantigens that met the prefiltering criteria (data not shown). Our computational pipeline, VACINUS, is mainly composed of peptide-MHC binding prediction and immunogenicity prediction using TCR information. The peptide-MHC binding prediction protocol VACINUS_pMHC_ is an ensemble model that utilizes 13 features (Supplementary Table 2). We initially considered 20 features and reduced them to 13 after recursive feature elimination while optimizing PRAUC (0.347 to 0.383 after feature selection). Considering both the F1 score and immunogenicity-positive neoantigen sensitivity, we selected the point with the highest sensitivity while maintaining the highest F1 score as our criterion for selection. This threshold, 0.14, represents the suitable agreement between the sensitivity and PPV (0.76 and 0.25, respectively).

**Fig. 2:**
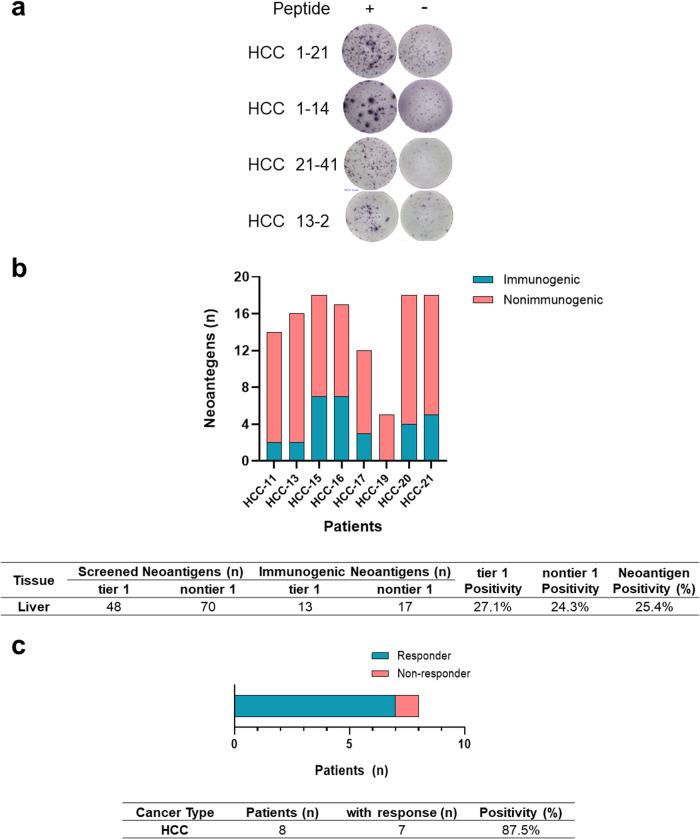
In vitro validation of the predicted neoantigens. **a** Representative images of IFN-γ ELISpots generated from PBMCs stimulated with peptide antigens. **b** Immunogenic and nonimmunogenic neoantigens from 8 patients with HCC. **c** Of the 8 patients with HCC, 7 (87.5%) exhibited immunogenicity for at least one neoantigen.

Subsequent validation of the VACINUS_pMHC_ workflow in a validation cohort of 8 patients with HCC identified a total of 118 neoantigens. The synthesized neoantigens, crafted as short peptides, underwent in vitro immunogenicity screening utilizing autologous PBMCs. Of the 118 neoantigens, 30 were confirmed to be immunogenic by in vitro immunogenicity screening (25.4%, Supplementary Table 3, Fig. 2a, b), which is relatively better than previously reported results^2,15^. The evaluation of VACINUS_pMHC_ on the TESLA dataset demonstrated a neoantigen selection sensitivity of 81.5% (Supplementary Fig. 2), surpassing the leading team’s achievement of 73.1% and outperforming the top 5 teams’ average sensitivity of 59%^21^.

### Development of VACINUS_TCR_ and in vitro validation

In pursuit of a comprehensive neoantigen prediction model grounded in a tumor-reactive TIL TCR-pMHC ternary complex, we conducted scRNA-seq on a collective pool of 202,575 CD8+ TILs obtained from the training cohort of 25 cancer patients for neoantigen prediction workflows (CRC: 6, GC: 11, HCC: 6, MN: 2) (Supplementary Fig. 3a). Further details can be found in the supplementary materials and methods. After quality control, a subset of 70,817 cells met the established criteria, with further selection narrowing to 25,411 CD8+ T cells expressing CD8 and excluding those expressing CD4. Through cluster-based T cell annotation of the aforementioned 25,411 cells, a subset comprising 14,592 cells was identified and designated putative tumor-reactive T cells. This subset constituted 57.4% of the total CD8+ T cell population. Notably, putative tumor-reactive CD8+ T cells encompass the following functional phenotypes: exhausted T cells, proliferative T cells, activated T cells, and effector T cells^15–18^ (Supplementary Fig. 3b). As previously shown^17^, the expansion indices of putative tumor-reactive T cells and the remaining T cell populations revealed a significant increase in clonality among the tumor-reactive T cells (*p* < 0.01, Supplementary Fig. 3c). The TCR β chain repertoire of these tumor-reactive T cells was characterized by sequencing highly variable CDR3 regions. pMTnet (v1.0)^20^ was utilized to predict the binding specificity between the CDR3β sequences of putative tumor-reactive T cells and pMHC complexes to establish VACINUS_TCR_. Neoantigens predicted to bind the TCR of putative tumor-reactive T cells are designated tier 1 neoantigens, and the remaining candidates are designated nontier 1.

We then tried to validate this workflow using a distinct dataset from 8 patients with HCC (Supplementary Fig. 4). Among the 99,331 CD8+ sorted TILs sequenced, 24,091 cells successfully met the quality control threshold. Within this refined subset, 21,961 cells were identified as CD8+ T cells. Notably, among the CD8+ T cell population, 12,650 cells were categorized as putative tumor-reactive T cells, constituting 57.6% of the total T cell population. The marker genes used to identify the CD8+ T cell subtypes were similar to those used in other studies^16,22,23^ (Supplementary Fig. 4a–c), and the gene expression levels for each subtype are listed in Supplementary Table 4. The putative tumor-reactive CD8+ T cells included exhausted T cells, proliferative T cells, activated T cells, and effector T cells (Supplementary Fig. 4d). The clonality observed in putative tumor-reactive T cells surpassed that in the other T cells, demonstrating statistical significance (*p* < 0.05) (Supplementary Fig. 4e). The 12,650 putative tumor-reactive T cells exhibited a repertoire of 2526 unique TCR clones. These unique TCR clone sequences were then leveraged for the selection of tier 1 neoantigens. From the VACINUS_pMHC_ workflow, a comprehensive total of 118 neoantigens were identified from a distinct cohort of 8 patients with HCC. Out of this pool, 48 neoantigens were predicted to bind with the TCRs of putative tumor-reactive T cells using pMTnet (v1.0)^20^. These neoantigens were subsequently designated tier 1. Of the 48 tier 1 neoantigens, 13 demonstrated immunogenicity (27.1%, in vitro validation for VACINUS_pMHC_), whereas 17 out of the 70 nontier 1 neoantigens showed immunogenicity (24.3%) (Fig. 2b, Supplementary Table 5). Among the 8 patients, 7 showed positive responses to one or more neoantigens, resulting in a response rate of 87.5% (Fig. 2c). The variability in neoantigen immunogenicity across patients is evident in these data. The number of tier 1 neoantigens ranged from 2 to 10 per patient, while the number of nontier 1 neoantigens ranged from 3 to 15. The positivity rates for immunogenicity showed considerable variability among the neoantigen candidates, ranging from 12.5% to 41.2% across different patients (Supplementary Table 5).

### Identification of tumor neoepitopes expressed by B16F10 tumors in vivo

Whole-exome sequencing (WES) and WTS were performed on three distinct B16F10 tumor tissues. Highly expressed nonsynonymous and indel mutations were identified through tumor–normal DNA analysis from splenocytes harvested from the same animals. Mutations observed in all three mice were considered neoantigen candidates (Supplementary Fig. 5a). A total of 510 nonsynonymous mutations expressing mutated alleles were identified. Should neoantigens be presented by MHC-I molecules on tumor cells to drive CD8+ T cell responses, the binding affinity of a peptide to MHC-I is the major criterion for identifying neoantigens. In total, we identified 186 potential neoepitopes from B16F10, representing 140 unique nonsynonymous/InDel mutations. Using VACINUS_pMHC_, a total of 30 candidate neoantigens were predicted from the B16F10 tumor model (Supplementary Fig. 5b, Supplementary Table 6).

To further enrich immunogenic neoepitopes, the TCR/pMHC interaction was considered based on the assumption that tumor-specific pMHC complexes are recognized by T cells and trigger an anticancer immune response in patients. We characterized the TCR β chain repertoire in putative tumor-reactive TILs by sequencing highly variable CDR3 regions. scRNA and TCR sequencing were performed on CD8+ TILs obtained from the B16F10 tumor model. We categorized a total of 6677 CD8+ T cells into five distinct clusters based on their gene expression profiles. These clusters included activated T cells, exhausted T cells, non-exhausted memory-like T cells, proliferative T cells, and NK-like T cells. Among the analyzed CD8+ T cells, three subtypes—exhausted T cells, proliferative T cells, and activated T cells—were designated putative target TILs, collectively constituting 70.4% (4,701 cells) of the total population (Supplementary Fig. 5c, d, Supplementary Table 7). Within the CD8 + TIL subset, the highest proportion was attributed to activated T cells, accounting for ~30%, followed by exhausted T cells, non-exhausted T cells, and proliferative T cells, each contributing ~20%. NK-like T cells comprised ~10% of the CD8+ TIL population (data not shown). The 30 candidate neoantigens were further divided into 2 categories, tier 1 and nontier 1, based on the TCR of the tumor-reactive TIL/pMHC interaction. Ten neoantigens were selected as tier 1 antigens in the B16F10 tumor model (Supplementary Table 6).

**Fig. 3:**
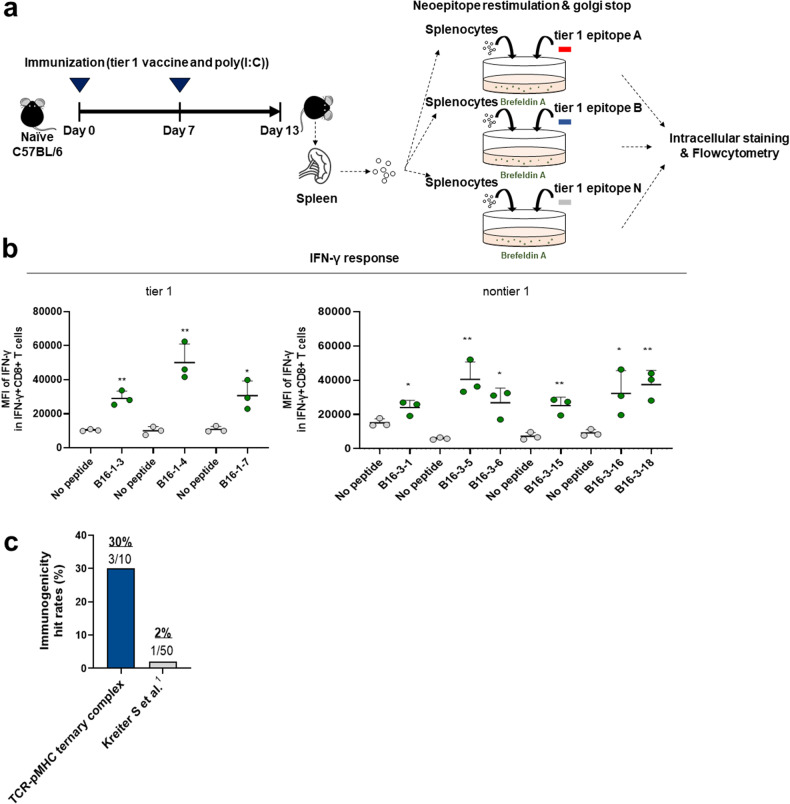
In vivo immunogenicity of neoantigen peptides. **a** Naive mice were vaccinated subcutaneously 2 times a week with synthetic long peptide (SLP) bearing tier 1 or nontier 1 neoantigen and adjuvant poly(I:C) and were sacrificed on Day 13. The experimental groups included those receiving poly(I:C) only or poly(I:C) combined with SLP. **b** Mean fluorescence intensity (MFI) of IFN-γ in CD8+ T cells determined by FACS. Splenocytes isolated from immunized mice were restimulated in vitro with the tested antigens and stained with CD8 and IFN-γ antibodies. **c** The hit rate of ‘VACINUS’ was confirmed by the estimation of immunogenicity after vaccination. The results were pooled, with *n* = 3 mice per group. *p* values are shown; statistical comparisons were performed using two-way ANOVA. *p < 0.05, **p < 0.01.

Synthetic long peptides (SLPs, 27 amino acids) targeting the 30 candidate MHC class I neoantigens were synthesized. The immunogenicity of each peptide was assessed by vaccinating non–tumor-bearing mice with 2 neoepitope peptides dissolved in sterile water with poly (I:C) as an adjuvant. Following the experimental plan, the mice were sacrificed, and splenocytes were extracted for further analysis. Splenocytes were then stimulated with 10 µg/ml short peptide neoantigen, and IFN-γ-producing CD8+ T cells were analyzed by flow cytometry (Fig. 3a). Neoantigens were considered immunogenic based on the mean fluorescence intensity (MFI) of IFN-γ+ CD8+ T cells compared to that of the background control without peptide stimulation (Fig. 3b). Immunogenicity was confirmed in three (B16-1-3-E1, B16-1-4-E1, and B16-1-7-E1) tier 1 neoantigens and in six (B16-3-1-E1, B16-3-5-E1, B16-3-6-E1, B16-15-E1, B16-3-16-E1, and B16-3-18-E1) nontier 1 neoantigens. Of the 10 tier 1 neoantigens predicted using our pipeline in the B16F10 tumor model, 3 (30%) demonstrated in vivo immunogenicity, which exceeded the predictive power reported in other studies (Fig. 3c)^24^.

The genes associated with these 9 in vivo immunogenic neoantigens were *Pcmtd1*, *D230025D16Rik*, *Nfrkb*, *Rpl13a*, *Pnp*, *Ctsd*, *Pask*, *Tbc1d5*, and *Hsf2*. Of the 9 neoantigens, 3 neoantigens overlapped with previously reported neoantigens with known immunogenicity in B16F10 cells, suggesting that these neoantigens exhibited the same characteristics as previously reported. The remaining 6 neoantigens with in vivo immunogenicity were novel predictions by the VACINUS platform (Supplementary Table 8).

Based on these observations, we chose to vaccinate tumor-bearing mice using immunogenic neoepitopes. Of the 9 neoantigens derived from B16F10 cells confirmed to have in vivo immunogenicity, 6 neoantigens, including 3 tier 1 (1–3, 1–4, 1–7) and 3 nontier 1 (3-1, 3–5, 3–6), were selected for in vivo antitumor efficacy testing.

### Neoantigen vaccination combined with anti-PD-1 therapy inhibited tumor growth

**Fig. 4:**
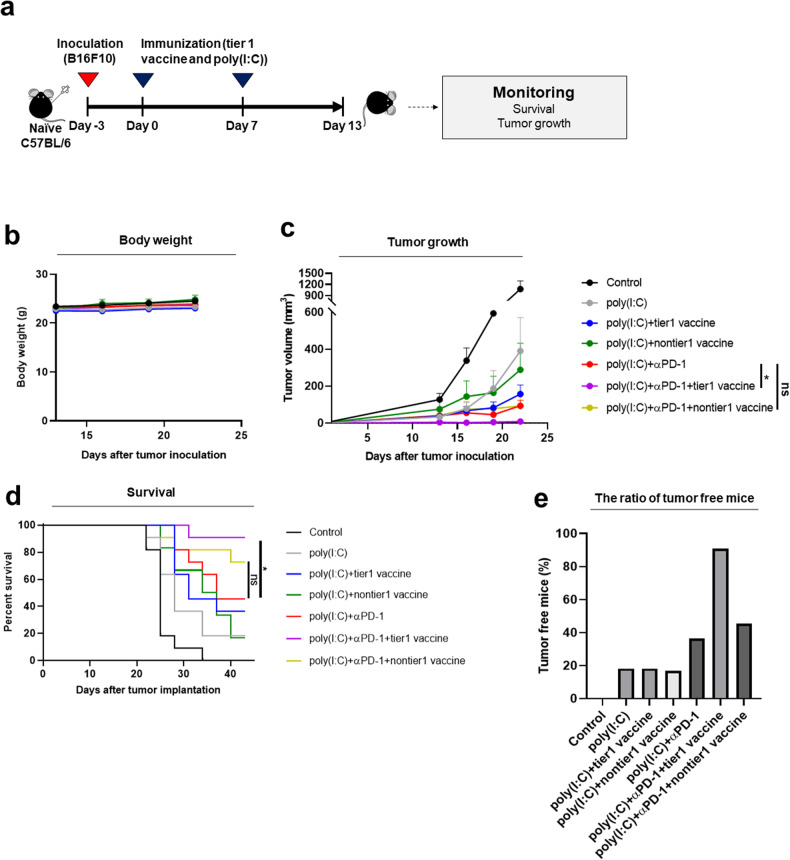
Antitumor effect of the tier 1 vaccine in a B16F10 mouse melanoma model. All mice were monitored for tumor growth and survival. **a** B16F10 tumor cells (2.5 × 10^5^ cells) were injected into the right flank subcutaneously. The synthetic long peptide (27-mer) vaccine was composed of a pool of 3 SLPs from tier 1 or nontier 1 epitopes. The anti-PD-1 was injected intraperitoneally 5 times every 3 days, and the vaccine was injected subcutaneously into the right flank 2 times a week 3 days after cancer cell inoculation. Seven groups of mice were used (*n* = 11 mice per treatment group): control, poly(I:C) alone, poly(I:C)+tier1 vaccine, poly(I:C)+nontier1 vaccine, poly(I:C)+anti-PD-1, poly(I:C)+aPD-1+tier 1 vaccine, and poly(I:C)+aPD-1+nontier1 vaccine combination. **b** Effects of various treatments on body weight. There were no significant differences in body weight throughout the entire experimental period. **c** Growth curves of subcutaneous tumors in B16F10 melanoma-bearing mice receiving the respective therapies. The tumor volume was measured on Days 13, 16, 19, and 22. **d** Survival curves of mice bearing B16F10 tumors after various treatments. The survival of the mice was measured until Day 45. **e** The percentage of mice that were tumor-free after various treatments.

We first investigated the efficacy of selected neoantigens in regulating tumor growth using the B16F10 tumor model. C57BL/6 mice were subcutaneously injected with 2.5 × 10^5^ B16F10 cancer cells, and neoantigen vaccination started 3 days later in the therapeutic setting shown in Fig. 4a. All 7 treatments were well tolerated, with no weight loss throughout the entire experimental period (Fig. 4b). Compared with the poly(I:C) treatment, monotherapy with tier 1 neoantigens did not significantly improve tumor growth (*p* = 0.239), and monotherapy with nontier 1 neoantigens showed no improvement in tumor growth (*p* = 0.968) (Fig. 4c, Supplementary Fig. 6a, b). However, combination treatment with tier 1 neoantigen and anti-PD-1 significantly reduced tumor growth (tier 1: *p* = 0.019), whereas combined treatment with nontier 1 neoantigen and anti-PD-1 was not equally effective (nontier 1: *p* = 0.986). Survival analysis using Kaplan‒Meier curves revealed a significant increase in survival in the combined treatment group with anti-PD-1+tier 1 neoantigen compared to the poly(I:C)+anti-PD-1 group (*p* = 0.027) (Fig. 4d). The mice in the anti-PD-1+nontier 1 neoantigen combination group had longer survival; however, the difference was not statistically significant (*p* = 0.235). No significant differences in survival were found between tier 1 (*p* = 0.161) or nontier 1 neoantigen (*p* = 0.497) compared to the poly(I:C) monotherapy. The combination of tier 1 neoantigen with anti-PD-1 therapy increased the percentage of mice rendered tumor-free (i.e., complete remission at 25 days after treatment). In the group treated with anti-PD-1 alone, 4 out of 11 mice were tumor-free, whereas, in the group treated with both anti-PD-1 and tier 1 neoantigen vaccination, 10 out of 11 mice were tumor-free (Fig. 4e).

### Tier 1 neoantigen-induced tumor growth inhibition is associated with increased numbers of neoepitope-specific CD8+ T cells

To further investigate the immune responses to tier 1 neoantigen peptides, C57BL/6 mice were injected subcutaneously with B16F10 melanoma cells (2.5 × 10^5^ cells per mouse) on Day −3 following the experimental schedule (Supplementary Fig. 7a). After the tumors were established, the mice were administered tier 1 neoantigen (SLP of 27-mer) + poly (I:C) adjuvant on Days 3 and 10. In addition, anti-PD-1 was injected intraperitoneally every 3 days for 2 weeks (100 µg per mouse each time). To assess the antitumor efficacy and toxicity of the tier 1 neoantigen vaccine, tumor size and body weight were measured on Day 16 after tumor inoculation. No changes in weight were observed across the 5 treatment groups throughout the entire experimental period (Supplementary Fig. 7b). Compared with PBS, the tier 1 neoantigen vaccine alone and anti-PD-1 combination therapy significantly reduced tumor growth (tier 1: *p* < 0.001, tier 1 + anti-PD-1: *p* < 0.001) (Supplementary Fig. 7c).

To examine the tier 1 neoantigen-specific T cell response, mice were sacrificed on Day 13, and splenocytes were isolated. We first assessed the antigen specificity of splenocytes against overlapping 9-mer peptides corresponding to the immunizing peptides. Splenocytes were subjected to restimulation with 10 µg/ml tier 1 neoepitopes, which were 9 amino acids in length. The percentage of CD8+ T cells secreting IFN-γ was quantified through FACS analysis. As illustrated in Supplementary Fig. 7d, discernible interferon responses were observed exclusively in the vaccinated groups, in contrast to the unvaccinated control groups. These data indicated that the tumor-reducing effect of control poly(I:C) treatment was not related to specific T cell activation but rather to the nonspecific innate immune response induced by the adjuvant.

**Fig. 5:**
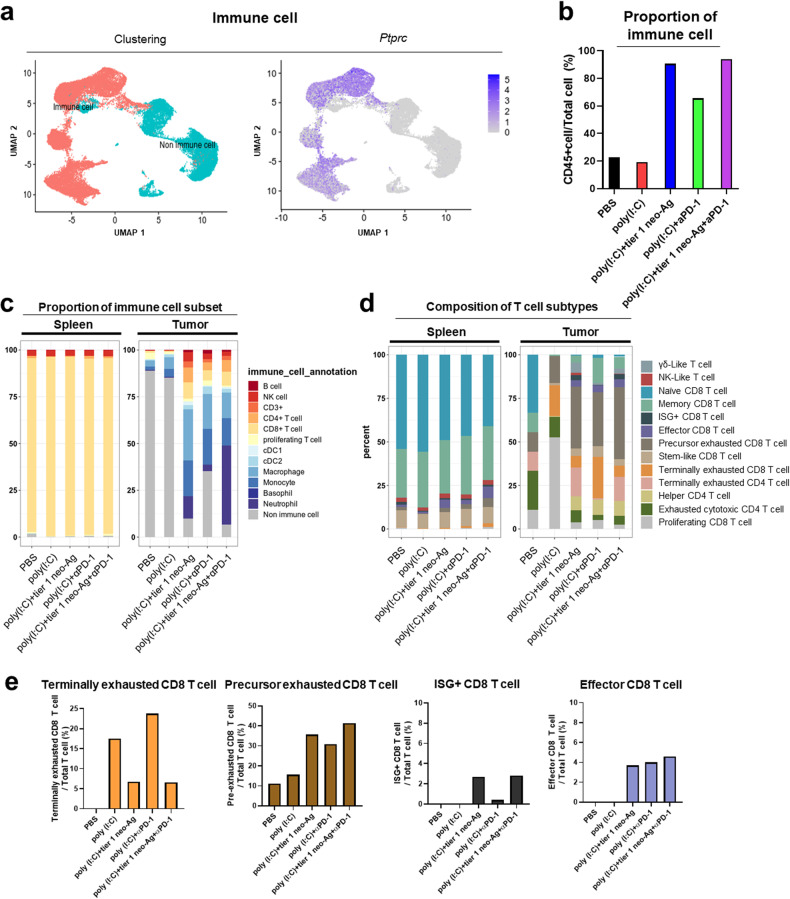
Induction of T cell immune responses by the tier 1 vaccine. The tumors from each treatment group were pooled (*n* = 5), dissociated and subjected to scRNA-seq. The spleens from each treatment group were pooled (*n* = 5) and sorted for antigen-specific CD8+ T cells using dextramer and anti-CD8 antibodies and subjected to scRNA-seq. ScRNA-seq data were pooled from 5 mice per group (*n* = 5). **a** Clustering and UMAP visualization of cells isolated from the spleen and tumor. UMAP visualization of CD45+ populations, colored. CD45+ cells were immune cells and CD45− cells were nonimmune cells. **b** The proportions of CD45+ cells among total tumor cells are shown according to treatment. **c** Proportion of immune cell subtypes based on gene markers. The five stacked bar graphs on the left show data from the spleen, and the five stacked bar graphs on the right show data from the tumor. **d** Proportions of T cell subsets among CD3+ T cells in the spleen and tumor are shown according to treatment. **e** Proportion of T cell subtypes according to treatment.

To elucidate the specific T cell subtypes and states contributing to the immune responses triggered by our vaccine, we conducted scRNA-seq and TCR-seq analyses on splenic CD8+ T cells that bind to the tier 1 epitope-dextramer, as well as on the entire tumor population. To explore the subtypes of T cells in greater detail, clustering of the obtained T cells was performed on a total of 43,670 scRNA-seq datasets derived from 4367 single-cell averages in each experimental group from the spleen and tumor. Comprehensive analysis of CD8+ T cells obtained from the spleen and tumor revealed both immune and nonimmune cells delineated by the expression of *Ptprc* (CD45+) (Fig. 5a). The proportion of CD45+ cells in the tumor was much greater in the vaccinated group than in the unvaccinated group (Fig. 5b). Both lymphoid and myeloid cell numbers were greater in the vaccinated group than in the unvaccinated group (Supplementary Fig. 8a). Subsequent to the initial analysis, a more comprehensive investigation was undertaken, resulting in the identification of 12 distinct immune subsets within the CD45+ cell population of the tumor. These subsets were characterized based on the expression profiles of established marker genes (Supplementary Table 9). Comparison of the proportions of immune cell subsets in the tumors between the vaccinated and unvaccinated groups revealed that both CD4+ and CD8+ T cells were increased in the vaccinated group (Fig. 5c). Additionally, natural killer (NK) cells, macrophages and monocytes accumulated in the tumors of the vaccinated groups (Supplementary Fig. 8b). Thirteen clusters of T cells were identified and annotated based on the expression of marker genes (Supplementary Fig. 8c, d, Supplementary Table 10). The compositions of the 13 subtypes of T cells are shown in a stacked bar graph (Fig. 5d). Analyses of T cell subtypes in the tumor showed that, except for terminally exhausted CD8+ T cells, T cells with proper functions, including pre-exhausted CD8+ T, ISG+ (interferon-stimulated genes)^25^ CD8+ T, and effector CD8+ T cells, were increased in the vaccinated group compared to the unvaccinated group (Fig. 5d, e). A substantial reduction in the percentage of terminally exhausted CD8+ T cells within the vaccinated group provides compelling evidence that either tier 1 vaccination specifically enhanced proliferation, thus increasing the percentage of precursor exhausted CD8+ T cells, or, more interestingly, tier 1 vaccination effectively hindered the progression of CD8+ T cells from a lightly exhausted state to a fully dysfunctional state (Fig. 5d, e). In the spleen, a greater percentage of effector CD8+ T cells was observed in the vaccinated group (Supplementary Fig. 8e).

**Fig. 6:**
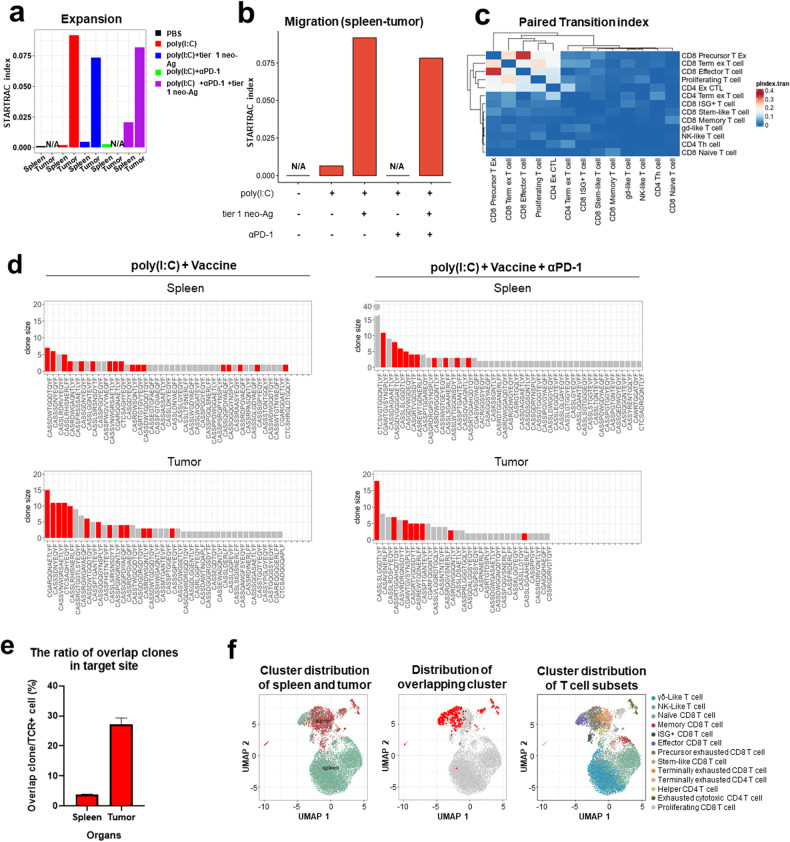
Antigen-specific CD8 T cell responses to the tier 1 vaccine. All single-cell data were analyzed with T cells pooled from the splenocytes and tumor cells of 5 mice. The proportions of the CD3+ T cell subsets were determined. The expansion, migration, and transition of T cell subsets were analyzed by STARTRAC tools. **a** Clonal expansion of T cells in the spleen or tumor was compared among groups. The proportions of T cell subtypes in the spleen and tumor are distinguished with different color bars. **b** The migration of T cells from the spleen to the tumor was calculated by STARTRAC in each treatment group. **c** Paired transitions between each subset of T cells are shown. **d** The TCR sequence from single-cell sequencing data was compared between splenocytes and tumor cells, and the matched TCR sequence is indicated in red. An exactly matched TCR sequence between splenocytes and tumor cells was considered an overlapping clone. TCR sequencing was performed on CD8+dextramer+ antigen-specific T cells sorted from the spleen and CD8+ T cells from the whole tumor. The data are not shown for the groups treated with PBS or poly(I:C) plus an anti-PD-1 antibody due to a lack of T cells. Clone size 1 was filtered out in the spleen, and a clone size >2 was used for TCR clone matching. **e** The frequency of overlapping clones in the spleen or tumor is shown in bars from the vaccinated group. **f** The proportion and distribution of overlapping clones in the spleen and tumor are shown in 2D UMAP, and T cell subtypes are indicated with colors.

To further investigate the complexities of T cell subsets in tumors following vaccination, T cell clonal expansion, migration dynamics, and T cell subset transitions were investigated using STARTRAC (Fig. 6a–c)^26^. The clonal expansion index ranged from 0 to 1, where the number indicates the degree of clonal expansion. The T cell expansion index was greater in the tumors than in the spleens of the vaccinated group (Fig. 6a). Along with the expansion index, the migration index in the vaccinated group was greater than that in the unvaccinated group (Fig. 6b). To assess whether the observed migration correlated with T cell functionality, we examined the expression of CXCR3, a chemokine receptor that is known to play a pivotal role in T cell trafficking and function^27,28^. CXCR3 expression was observed only in the vaccinated group, supporting the prevalence of functionally active migrating T cells (Supplementary Fig. 9a). When T cell plasticity was examined using the transition index, the most frequent transition was found between effector CD8+ T cells and pre-exhausted CD8+ T cells, followed by pre-exhausted CD8+ T cells and terminally exhausted CD8+ T cells (Fig. 6c). The expression of IFN-γ or Granzyme B was greater in the vaccinated group than in the unvaccinated group, verifying the effector function of T cells in tumors (Supplementary Fig. 9b, c).

We compared the extent of TCR repertoire overlap between CD8+ TILs and splenic T cells using single-cell TCR sequencing. The hypervariable CDR3β is unique to individual T cell clones and can, therefore, be used to monitor the dynamics of T cell clonality. Due to the high clonal variability of the splenic TCR, 2> clones were selected. Clones overlapping with TILs were identified only in the vaccinated group (Fig. 6d, Supplementary Fig. 10a). In the vaccinated group, more than 25% of the T cells that infiltrated the tumor overlapped with those in the spleen (Fig. 6e, Supplementary Fig. 10b). These overlapping clones were mainly present in effector/exhausted CD8+ T cells (Fig. 6f).

## Discussion

In this study, we developed neoantigen prediction workflows, VACINUS_pMHC_ and VACINUS_TCR,_ based on the neoepitope presentation on the patient’s MHC class I molecules and the interaction between the patient’s putative tumor-reactive CD8+ TIL TCR and neoepitope-MHC recognition, respectively, and validated the predicted neoantigens in vitro and in vivo. VACINUS_pMHC_ was effective at predicting neoantigens, showing a neoantigen selection sensitivity of 81.5% using the TESLA dataset, which was higher than the 73.1% sensitivity achieved by the leading TESLA team^21^. Neoepitope prediction algorithms have predominantly been based on mutation selection and binding to patient MHC molecules^2,4,5,29^. Since T cells recognize neoantigen peptides presented on MHC class I molecules via TCRs, the interaction between TCRs and pMHCs is critical. Therefore, we prioritized immunogenic neoantigens by assessing the binding specificity between TCRs (CDR3β sequences) and HLA based on peptide sequences using pMTnet (v1.0)^20^. Neoantigens were classified into either tier 1 or nontier 1 according to the calculated binding affinity of the TCR of tumor-reactive TILs and pMHC complexes. Tier 1 vaccination reduced tumor growth, and when combined with anti-PD-1 therapy, it led to complete remission of the tumor. Tier 1 monotherapy and the combination treatment were superior to the nontier 1 vaccination (Fig. 4).

The tier 1 vaccine was immunogenic and elicited antitumor effects, whereas the activity of the nontier 1 vaccine was not comparable to that of the control, suggesting that TCR-pMHC binding is essential for eliminating cancer cells and for neoantigen-based immunotherapies. Previous studies have shown favorable antitumor immune responses in mouse models and in melanoma and glioblastoma patients without the use of immune checkpoint inhibitors^2,3,5^. In our study, monotherapy with tier 1 neoantigens improved tumor growth compared to that in the poly(I:C) group, although the difference was not statistically significant. In several previous reports in which neoantigen peptide alone was not effective^30^, poly(I:C) was also used as a vaccine adjuvant to enhance innate immunity to trigger adaptive immune responses. Treatment with poly(I:C) alone strongly inhibited tumor growth and increased survival. Additionally, treatment with the adjuvant poly(I:C) without the addition of peptides resulted in a fairly good antitumor response, which is a typical characteristic of B16F10 melanoma cells, and increased variability in responsiveness. Previous studies used up to 20 peptides in 4–5 pools^3,5^ compared to the 3 peptides used in our study. All these parameters might have underestimated the efficacy of our VACINUS platform. Moreover, the number of CD8+ T cells producing effector cytokines increased only in the vaccinated group and not in the control poly(I:C)-only group. These data indicated that the tumor-reducing effect of poly(I:C) treatment alone was not related to specific anticancer T cell activation but rather to nonspecific innate immune activation.

The prediction of highly cancer-specific TCRs and agonistic neoantigens reflects the way our body selects the self-MHC-restricted T cell repertoire in the thymus during development (i.e., the characteristic nature of thymic positive selection). After thymic positive selection, the repertoire of high-affinity antigen-specific T cells is reduced compared to that of low- to intermediate-affinity T cells. Thus, the precise selection of neoantigens through an algorithm based on the interaction of tumor-reactive TIL TCRs with pMHC molecules will not only stimulate a specific anticancer immune response but also reduce adverse immune-related events.

The limitations of our study include the following: (1) Additional studies are warranted to elucidate why nontier 1 vaccination failed to control tumor growth. (2) Only the publicly available pMTnet tool was utilized for predicting TCR-pMHC binding. A more precise model is needed to improve TIL TCR-pMHC binding prediction.

Numerous challenges must be overcome for personalized neoantigen-based cancer vaccines to progress and become established as mainstream therapeutic approaches. Of all the challenges to be confronted, the swift and accurate prediction of immunogenic neoantigens is imperative because in vivo evaluation of neoantigens derived from in silico analyses is excessively time-consuming. Our study confirmed that the prediction of neoantigens based on a ternary complex of tumor-reactive TIL and the pMHC can enhance the identification of immunogenic neoantigens.

### Supplementary information

SUPPLEMENTARY INFORMATION

Supplementary Table 4

Supplementary Table 7

Supplementary Table 10

## Data Availability

All data relevant to the study are included in the article or uploaded as supplementary information. The data are available upon reasonable request (woongyang.park@kr-geninus.com).
