# Supplementary material for: The identification of effective tumor-suppressing neoantigens using a tumor-reactive TIL TCR-pMHC ternary complex
Source: Exp Mol Med. 2024 Jun 12;56(6):1461–71. doi: 10.1038/s12276-024-01259-2 (PMC11263684; doi:10.1038/s12276-024-01259-2)
Supplement: Supplementary file 1 — SUPPLEMENTARY INFORMATION [file 12276_2024_1259_MOESM1_ESM.pdf]

SUPPLEMENTARY INFORMATION

The identification of effective tumor-suppressing neoantigens using a tumor-reactive TIL TCR-pMHC ternary complex.

Sang Hoon Kim,[1,*] Bo Ryeong Lee,[1,*] Sung-Min Kim,[1,*] Sungsik Kim,[1] Min-seok Kim,[1] Jaehyun Kim,[2] Inkyu Lee,[2,3] Hee-Soo Kim,[1] Gi-Hoon Nam,[2,4] In-San Kim,[3,5] Kyuyoung Song,[1] Yoonjoo Choi,[6,#] Dong-Sup Lee,[7,#] Woong-Yang Park [1,8,9,#]

The file contains Supplementary materials and methods, Supplementary figures 1−10, and Supplementary tables 1−10

Table of Contents

Supplementary materials and methods………………………………………………………………. 4

SI References……………………………………………………………………………………. 11

Supplementary figures

Supplementary figure 1. Sensitivity for the prefiltration criteria using immunogenic neoantigens from TESLA and in-house dataset……………………………………………………………………………. 13

Supplementary figure 2. Evaluation of VACINUS$_{pMHC}$ using the TESLA dataset and comparison with the top-5 teams' neoantigen detection sensitivity………………………………………………………………… 14

Supplementary figure 3. Single-cell analysis of CD8+ TILs from 25 cancer patients for the construction of a neoantigen selection model ……………………………………………………………………............15

Supplementary figure 4. Single cell analysis of CD8+ TILs from 8 patients with HCC………………………17

Supplementary figure 5. Neoantigens predicted from B16F10 tumor model and analysis of CD8+ TILs…….19

Supplementary figure 6. *In vivo* anti-tumor efficacy of candidate neoantigens……………………………….21

Supplementary figure 7. Antigen-specific immune responses after vaccination with tier 1 neoantigen………22

Supplementary figure 8. The profile of leukocytes and T cells after vaccination with tier 1 neoantigen……...24

Supplementary figure 9. Analysis of functional T cells after vaccination with tier 1 neoantigen……………..26

Supplementary figure 10. Overlapping CD8+ T cell clones in unvaccinated groups………………………….27

Supplementary tables

Supplementary table 1. The bioinformatic toolsets used in $VACINUS_{pMHC}$ and $VACINUS_{TCR}$………………28

Supplementary table 2. Twenty features considered for the ensemble model…………………………………29

Supplementary table 3. The validation of $VACINUS_{pMHC}$ workflow using immunogenic in vitro screening from PBMCs of HCC patients…………………………………………………………………...……...30

Supplementary table 4. DEG analysis of tumor-reactive TILs in 8 patients with HCC………………..……..31

Supplementary table 5. Validation of predicted neoantigens in 8 patients with HCC…………………………32

Supplementary table 6. Thirty neoantigens identified in mouse B16F10 tumor model using $VACINUS_{pMHC}$ and 10 tier 1 neoantigens……………………………………………………………………………...33

Supplementary table 7. DEG analysis of TILs in B16F10 tumor……………………………………………..34

Supplementary table 8. Neoepitopes with positive immunogenicity………………………………………….35

Supplementary table 9. Marker genes for specific immune cell type………………………………………….36

Supplementary table 10. DEG analysis of T cell subsets in B16F10 bearing mice after tier 1 vaccination……37

SUPPLEMENTARY MATERIALS AND METHODS:

Patient materials

Fresh frozen tumor tissue was obtained during the surgical procedure and collected in RPMI 1640 (Welgene, Daegu, Korea) supplemented with 10% fetal bovine serum (FBS, Gibco). Peripheral blood (PB) from the same patient was collected up to 20 mL in EDTA tubes (BD, USA). A portion of the tumor tissue was excised for whole exome sequencing (WES) and whole transcriptome sequencing (WTS), while the remaining tissue was used for single-cell RNA-sequencing (scRNA-seq) following dissociation. The tumor tissues were enzymatically dissociated into single-cell suspensions using the tumor dissociation kit (Miltenyi Biotec, Germany). CD8 T cells were isolated from the single-cell suspensions using EasySep human CD8+ T cell isolation kit (STEMCELL Technologies, Canada). The isolated CD8 T cells were used for single cell RNA and TCR sequencing. PB was used for WES and *in vitro* immunogenicity screening assay.

Cell culture

B16F10 cells (ATCC cat no. CRL-6475, USA) were cultured in high glucose DMEM (Hyclone, USA) supplemented with 10% FBS (Invitrogen, USA) and 1% antibiotic/antimitotic solution (Invitrogen). All cells were cultured at 37°C in a humidified atmosphere containing 5% CO_2.

WES, WTS, scRNA, scTCR sequencing

Genomic DNA samples (33 matched blood and tumor tissue) underwent library construction using the Twist Human Core Exome kit (Twist Bioscience, USA). The captured DNA libraries were sequenced with paired end reads of 150 bp on a NovaSeq6000 or NextSeq550 (Illumina, USA).

RNA capture with poly-A tail was performed using magnetic Oligo(dT) beads. RNA integrity was measured using Agilent 4200 TapeStation. The RNA libraries were constructed using the Illumina Stranded mRNA Prep Kit (Illumina). These libraries were sequenced with paired end reads of 150 bp on a NovaSeq6000 or NextSeq550.

The scRNA-seq libraries were prepared using the Chromium Next GEM Single Cell 5′ Kit v2 (Dual Index) of Chromium platform (10x Genomics, USA) following the manufacturer’s instruction. cDNA library quality

was determined using an Agilent Bioanalyzer (Agilent, USA). The scRNA libraries were sequenced on the Illumina NovaSeq 6000 sequencing platform using the following read lengths: read 1:50; i7 index:10; i5 index:10; read 2:100.

The scTCR libraries were enriched via PCR amplification using a Chromium Single-cell V(D)J library (v1.0 Chemistry). Around 12,000 cells were loaded per sample, with the targeted cell recovery of 8,500 cells according to the protocol. Single cells were isolated and lysed, mRNA was converted into barcoded cDNA through reverse transcription using provided reagents (10x Genomics). 13 PCR cycles were used to amplify cDNA and generated barcoded cDNA libraries for single cell 5`library. Part of the amplified cDNA was target-enriched for TCRs and V(D)J library was obtained according to manufacturer protocol. cDNA library quality was determined using an Agilent Bioanalyzer (Agilent). Barcoded libraries (included VDJ libraries) were pooled and sequenced with paired end on the Illumina Novaseq6000, using the following read lengths: read 1:50; i7 index:10; i5 index:10; read 2:100.

Single cell data processing

The FASTQ sequencing reads were processed using Cell Ranger version 5.0.0 (10x Genomics) and the GRCh38 human transcriptome reference for alignment. Data preprocessing was performed using Seurat v4.3.0[1], applying quality control criteria to filter out cells with a mitochondrial gene percentage greater than 20%. Only T cells detected in the TCR sequence from scTCR sequencing data were retained for downstream analysis. Specifically, T cells with CD8 expression without CD4 expression were selected for further analysis. Then, the raw count data were normalized, and scaling was performed by regressing out the mitochondrial gene percentage. Principal component analysis (PCA) was conducted using the 3,000 most variable features by Seurat. To avoid clonotype bias, TCR genes were removed from the variable features. Clusters were manually annotated using CD8+ T cell subtype markers to identify specific subtypes. We analyzed the clonal expansion of putative tumor-reactive TILs using the STARTRAC package[2].

Mapping, data processing and gene expression quantification

We used the Burrows-Wheeler Aligner MEM algorithm BWA-MEM (v0.7.17)[3] for WES and STAR aligner (v2.7.8a)[4] for WTS data to align to the reference genome (hg38). Alignment and data processing were carried

out following the Genome Analysis Toolkit (GATK) Best Practices guidelines for data pre-processing[5]. Transcriptome-level expression profiling was performed using the RSEM (RNA-Seq by Expectation-Maximization, v1.3.1) tool[6].

Somatic variant calling

Somatic variants, including single nucleotide variants (SNVs) and insertions/deletions (Indels), were identified using three different mutation callers, Strelka (v2.9.2)[7], Seurat (v2.5)[8], and Mutect2 (from the GATK v4.2.0.0)[9]. The resulting variants were then filtered using the GATK workflow, with the additional filtering criterion that gene expression profiling was utilized to filter variants based on their variant allele frequency (VAF). The Ensemble Variant Effect Predictor (VEP, v104.3)[10] was used for the analysis, annotation, and filtering of variants.

***In silico* HLA typing**

The 4-digit human leukocyte antigen (HLA) class I typing was performed using OptiType (v1.3.2)[11], a novel HLA genotyping algorithm based on integer linear programming, and the previously mapped DNA sequencing data from the tumor and blood samples.

***in vivo* Proof-of-Concept (PoC) of VACINUS platforms [$\text{VACINUS}_{\text{pMHC}}$ + $\text{VACINUS}_{\text{TCR}}$] for optimized selection of immunogenic neoantigen**

As the binding affinities between peptides and murine MHC class I molecules predicted higher compared to those in humans, our selection criteria for neoantigens have been adjusted. Criteria for selection of neoantigens include a binding affinity threshold of less than 200 nM for H2-Kb-peptide and less than 400 nM for H2-Db-peptide interactions.

Key marker genes used were different from those of humans. In mice, tumor-reactive TILs included activated, exhausted, and proliferative T cells, as determined by the expression levels of *Gzma* and *Gzmk*, *Tnfrsf9* and *Pdcd1*, and *Mki67* and *Pcna*, respectively.

As pMTnet does not support the mouse tumor genomics data, we needed an equivalent model for the murine system. We obtained the actual pMHC-TCR structures from the RCSB PDB database of X-ray crystallography

experimental data (https://www.rcsb.org/). TCRpMHCmodels (v1.0)[12] which takes the amino acid sequence of pMHC and TCR as input, was used to predict protein structure models. We then used FoldX (v5.0)[13] AnalyzeComplex to calculate the energy parameters for the pMHC-TCR protein structure. A logistic regression algorithm model was developed using the energy parameter values of protein structures to predict the binding between TCR and pMHC complexes. Using this model, the energy parameter values for tumor-reactive TILs TCR and pMHC interactions were calculated. Based on the results, we categorized TCR-pMHC pairs with a model score of 0.7 or higher as tier 1 neoantigens.

Peptides

Synthetic 9-10mer and 27mer peptides covering tumor-specific mutations were synthesized to > 90%, 4 mg per neoantigen by Biostem (South Korea). Peptides were dissolved in distilled water (DW) or DMSO to create a stock solution with a concentration of 20 mM. A working solution of 1 mM was prepared by diluting the stock solution 20-fold. The stock solution was stored in a deep-freezer, while the working solution was kept refrigerated for up to one month.

PBMCs isolation

PBMCs (peripheral blood mononuclear cells) were isolated from PB of HCC patients using Lymphoprep (STEMCELL Technologies) following the manufacturer's recommendations and cryopreserved until neoantigen synthetic peptides become available for *in vitro* immunogenicity screening.

***In vitro* immunogenicity screening**

Cryopreserved PBMCs from patients were thawed and suspended in AIM-V (Thermo Fisher, USA) supplemented with 10% human AB serum (Merck, USA). PBMCs were washed with PBS and centrifuged at 500g at RT for 10 minutes. After removing the supernatant, the pellet was resuspended in thawing media. Cells were treated with DNase (10mg/ml, STEMCELL Technologies) for 4 to 6 hours at 37°C in a humidified 5% CO_2 incubator, followed by harvesting under the same conditions. Cells were cultured at a density of 3×10^5 cells per well in a 96-well plate and treated with a single neoantigen peptide (10 μg/ml, 9~10 mer). In addition, each well was treated with anti-PD-1 antibody (5 μg/ml, Biolegend, USA). The cells were cultured

for 16 days and were treated with IL-2 (10 U/ml, PeproTech, USA) and IL-15 (5 ng/ml, PeproTech) cytokines on days 3, 6, 9, and 12. If the media color changed to yellow, half of the culture media was replaced with fresh media. On day 16, the human IFN-γ ELISpot PRO kit (ALP) (MabTech, Sweden) was used to determine the amount of cytokine-secreting T cells. T cells were seeded at 50,000 cells per well in a ELISpot plate followed by stimulation with individual neoantigen peptide (10 μg/ml) for 22 hours. All tests were performed in triplicate and included DMSO (0.05%) as negative controls and PHA (phytohemagglutinin) (Thermo Fisher) (1.25 mg/ml) as positive controls. Spots were visualized with a biotinylated-anti IFN-γ antibody following the manufacturer's instructions. Plates were scanned using an AID Classic ELISpot Reader (Germany). T cell responses stimulated with neoantigen peptides were compared to those treated with DMSO using a Mann-Whitney U test.

Statistics

Prism 8 (GraphPad) and SPSS were employed for statistical analysis. One-way analysis of variance (ANOVA) followed by Tukey's post hoc test was applied to assess differences among more than two groups. Kaplan-Meier analysis with the log-rank test was used to evaluate survival benefit. The error bars represent the standard error of the mean (SEM). Statistical significance was determined at p values < 0.001 and < 0.05.

Generation of B16F10 tumor bearing mice

All studies utilized male C57BL/6 mice of 6 weeks old. C57BL/6 were purchased from Orient Bio (South Korea). All mice were maintained in a specific pathogen–free (SPF) animal room at the Korea Institute of Science and Technology (KIST). All animal experiments were performed in accordance with the guidelines of the Institutional Animal Care and Use Committee (IACUC) of KIST. All mice were stabilized for at least one week prior to the experiment. Tumors were induced by injecting 1 x 10^6 cells of B16F10 subcutaneously in the right flanks of mouse. Tumor volume and body weights were measured every 4 days. Each experimental group comprised five mice housed together in a single cage. The mice were monitored daily for the duration of the experiment.

Acquiring the B16F10 tumor tissues and tail tips for VACINUS platform

When tumors reached a size of 100 mm^3, tumor tissues from B16F10 tumor-bearing mice were harvested. For control samples, approximately 1 cm of the tail tip was excised from normal C57BL/6 mice. These control samples provided a baseline comparison for the tumor tissues. The harvested tumor tissues and control tail tip samples were collected on the same day of extraction. The harvested tumor tissues were enzymatically dissociated into single-cell suspensions using the tumor dissociation kit (Miltenyi Biotec) and the gentleMACS™ Octo Dissociator (Miltenyi Biotec). The dissociated single-cell suspensions from tumor tissues were filtered through a 40 μm strainer to remove large debris. Erythrocytes were then lysed using an RBC lysis buffer (BioLegend). After removing dead cells using the specific kit (Miltenyi Biotec), CD8 T cells were magnetically enriched from the single-cell suspensions using CD8 T cell microbeads (Miltenyi Biotec). The purity of isolated CD8 T cells was confirmed by CD8 FACS analysis. The purified CD8 T cells were suspended in RPMI1640 media (Welgene) containing 10% FBS (Gibco), and subsequently used for scRNA-seq.

Immunization of mice with selected neoepitopes (27-mer peptide)

Male C57BL/6 mice, aged 6–8 weeks and matched in age, were injected subcutaneously with selected neoantigens. Each flank received a combination of the selected neoantigens (100 μg) and adjuvant (50 μg poly(I:C) (InvivoGen, France) in PBS, with a total of 100 μg poly(I:C) per mouse. The number of neoantigen groups was 15, with 3 mice per neoantigen, resulting in a total of 45 mice. Mice were immunized on day 0 and again on day 7. An adjuvant-only control group received 100 μg poly(I:C) injection without neoantigens. The injection solution was prepared by mixing a 10 mg/ml stock solution (10 μl) with a 1 mg/ml stock solution (50 μl), and diluting with PBS (140 μl) to a total volume of 200 μl per flank.

Immunogenicity of selected neoantigens (9-10 mer peptide)

On day 13, spleens were extracted from the immunized mice. A total of 2×10^6 splenocytes were incubated with 10 ug/ml CD8+ T cell epitopes of 9-10 mer of the injected neoantigens, along with protein transport inhibitors: 1:1,500 Monensin (Thermo Fisher Scientific), 1:1,000 Brefeldin A (BD Biosciences), 5μg/ml DNase I solution (GenDEPOT) for 6 hours. As a positive control, a cell stimulation cocktail (40.5 uM phorbol

12-myristate 13-acetate (PMA) and 670 uM ionomycin in ethanol (eBioscience, USA) was used. For a negative control, RPMI media was added to fill the appropriate volume for each group.

The immunogenicity of each neoantigen was evaluated by intracellular staining (ICS) of IFN-γ using the LIVE/DEAD™ Fixable Near-IR Dead Cell Stain Kit (Invitrogen), fixation/ permeabilization solution kit (BD), and antibodies specific for CD45.2, CD3, CD8, and IFN-γ (BioLegend). The percentage of IFN-γ-producing CD8+ T cells was analyzed using flow cytometry.

Therapeutic efficacy of neoantigens

Male C57BL/6 mice, aged 7-8 weeks, were subcutaneously injected with B16F10 cancer cells (2.5 x 10^5 cells) on the flank. The combination of 3 peptide neoantigens of the same tier (100 μg each) were mixed with 100 μg poly(I:C) in PBS for subcutaneous injection. The initial injection was administered when the average size of the cancer reached 30-70 mm^3, followed by a second injection after 7 days. Furthermore, intraperitoneal injections of aPD-1 (200 μg/mouse) were given a total of 5 times at 3-day intervals, starting from the first injection. The study included 7 treatment groups, each consisted of 7 mice, resulting in a total of 49 mice. Mice were monitored for tumor size, body weight, and survival rate at three-day intervals. Mice were sacrificed if the tumor size exceeded 2000 mm^3. The percentage of tumor free was assessed in mice by absence of palpable tumor on side flank on day 25 post tumor cell inoculation. The treatment groups for B16F10 neoantigens included the following: G1, the non-treated group; G2, the poly(I:C)-only group; G3, the poly(I:C) + tier1 vaccine group; G4, the poly(I:C) + nontier1 vaccine group; G5, the aPD-1 + poly(I:C) group; G6, the aPD-1 + poly(I:C) + tier 1 vaccine group; and G7, the aPD-1 + poly(I:C) + nontier1 vaccine group.

Neo-epitope specific CD8 T cell sorting

The acquisition of tier1-specific CD8 T cells from the spleen was performed using dextramers (Immudex, Denmark). Neoepitope-specific dextramers were assembled with peptides for mouse MHC, composed of B16-1-3 (VSFAPLVQL)-H2 Kb and B16-1-4 (CIIRNVQVL)-H-2 Db, encoding Pcmtd1 and D230025D16Rik, respectively.

The tier 1-specific CD8 T cells were sorted from the pooled splenocytes, stained with CD3 (BV421 anti-

mouse), CD8a (FITC anti-mouse), Dextramers (B16-1-3_PE, B16-1-4_APC), and Live/Dead (L34975; APC-A750 anti-mouse). They were then sorted by flow cytometry on a BD FACSAria III after enrichment for CD8 T cells using the CD8a T Cell Isolation Kit (Miltenyi Biotec).

SI REFERENCES

1. Hao, Y. et al. Integrated analysis of multimodal single-cell data. *Cell* **184**, 3573–3587.e29 (2021).
2. Zhang, L. et al. Lineage tracking reveals relationships of T cells in colorectal cancer. *Nature* **564**, 268–272 (2018).
3. Li, H. & Durbin, R. Fast and accurate short read alignment with Burrows-Wheeler transform. *Bioinformatics* **25**, 1754–1760 (2009).
4. Dobin, A. et al. STAR: ultrafast universal RNA-seq aligner. *Bioinformatics* **29**, 15–21 (2013).
5. McKenna, A. et al. The genome analysis toolkit: a MapReduce framework for analyzing next-generation DNA sequencing data. *Genome Res.* **20**, 1297–1303 (2010).
6. Li, B. & Dewey, C.N. RSEM: accurate transcript quantification from RNA-Seq data with or without a reference genome. *BMC Bioinformatics* **12**, 323 (2011).
7. Saunders, C.T. et al. Strelka: accurate somatic small-variant calling from sequenced tumor-normal sample pairs. *Bioinformatics* **28**, 1811–1817 (2012).
8. Christoforides, A. et al. Identification of somatic mutations in cancer through Bayesian-based analysis of sequenced genome pairs. *BMC Genomics* **14**, 302 (2013).
9. Cibulskis, K. et al. Sensitive detection of somatic point mutations in impure and heterogeneous cancer samples. *Nat. Biotechnol.* **31**, 213–219 (2013).
10. McLaren, W. et al. The Ensembl Variant Effect Predictor. *Genome Biol* **17**, 122 (2016).
11. Szolek, A. et al. OptiType: precision HLA typing from next-generation sequencing data. *Bioinformatics* **30**, 3310–3316 (2014).
12. Jensen, K. K. et al. TCRpMHCmodels: Structural modelling of TCR-pMHC class I complexes. *Sci. Rep.* **9**, 14530 (2019).
13. Delgado, J., Radusky, L.G., Cianferoni, D. & Serrano L. FoldX 5.0: working with RNA, small molecules

and a new graphical interface. *Bioinformatics* **35**, 4168–4169 (2019).

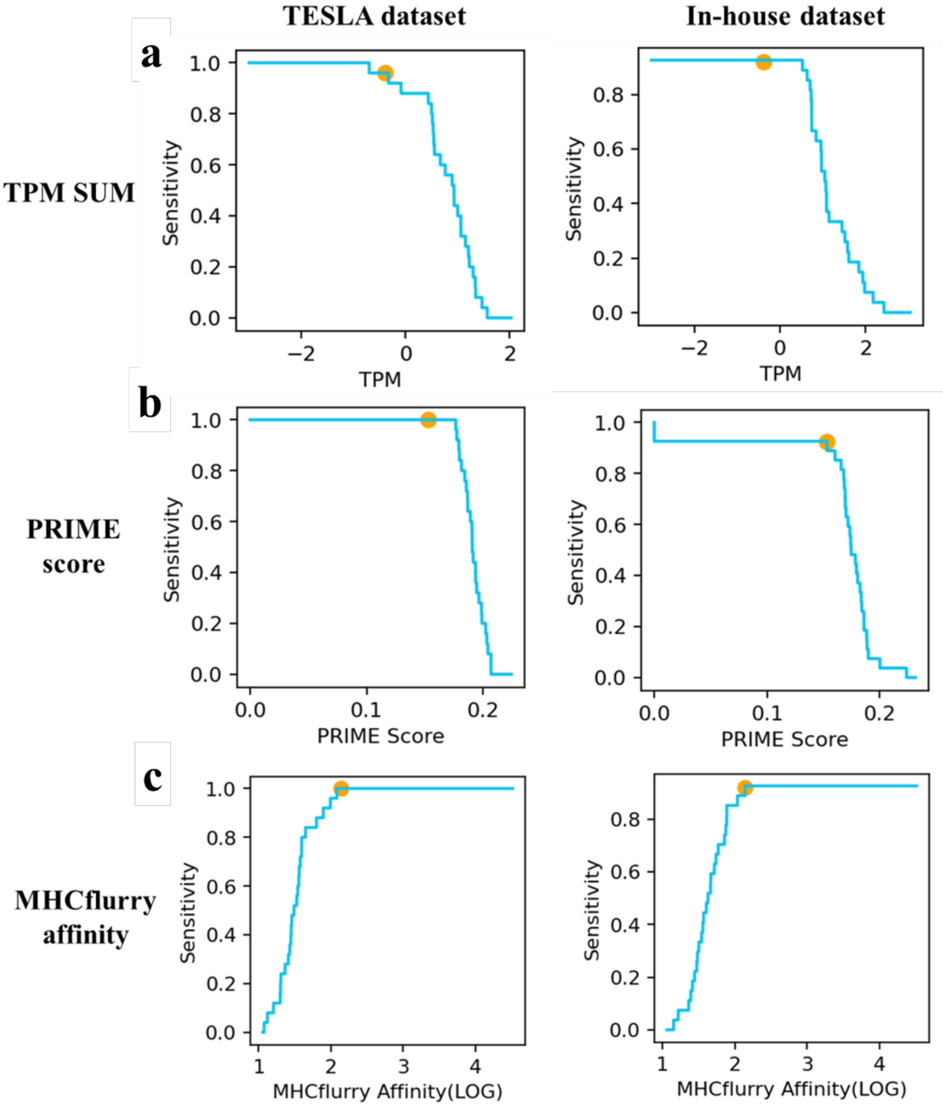

Supplementary Fig. 1. Sensitivity for the prefiltration criteria using immunogenic neoantigens from TESLA and in-house dataset. a Sensitivity-TPM graphs. The orange dot corresponds to the prefiltration condition of log 0.4 in the TPM sum value. **b** Sensitivity-PRIME score graphs. The orange dot corresponds to the prefiltration condition of 0.847 in the PRIME score. **c** Sensitivity-MHCflurry affinity graphs. The orange dot corresponds to the prefiltration condition of 140nM in the MHCflurry affinity.

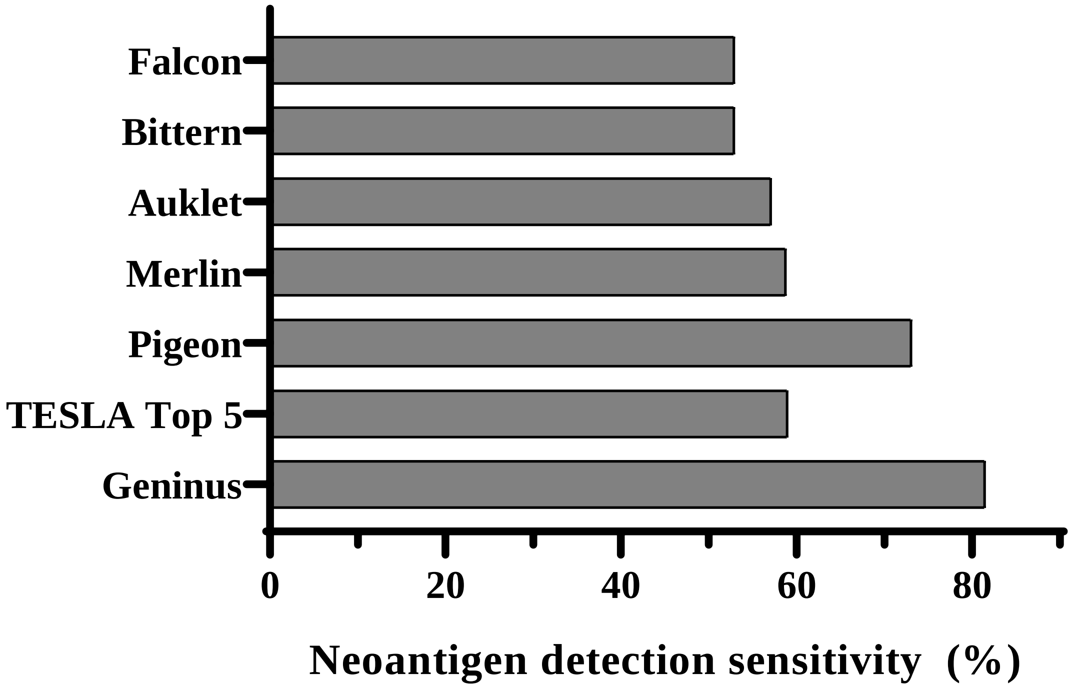

Supplementary Fig. 2. Evaluation of VACINUS$_{pMHC}$ using the TESLA dataset and comparison with the top-5 teams' neoantigen detection sensitivity. Data from 3 patients with melanoma and 2 patients with NSCLC in the TESLA dataset were used.

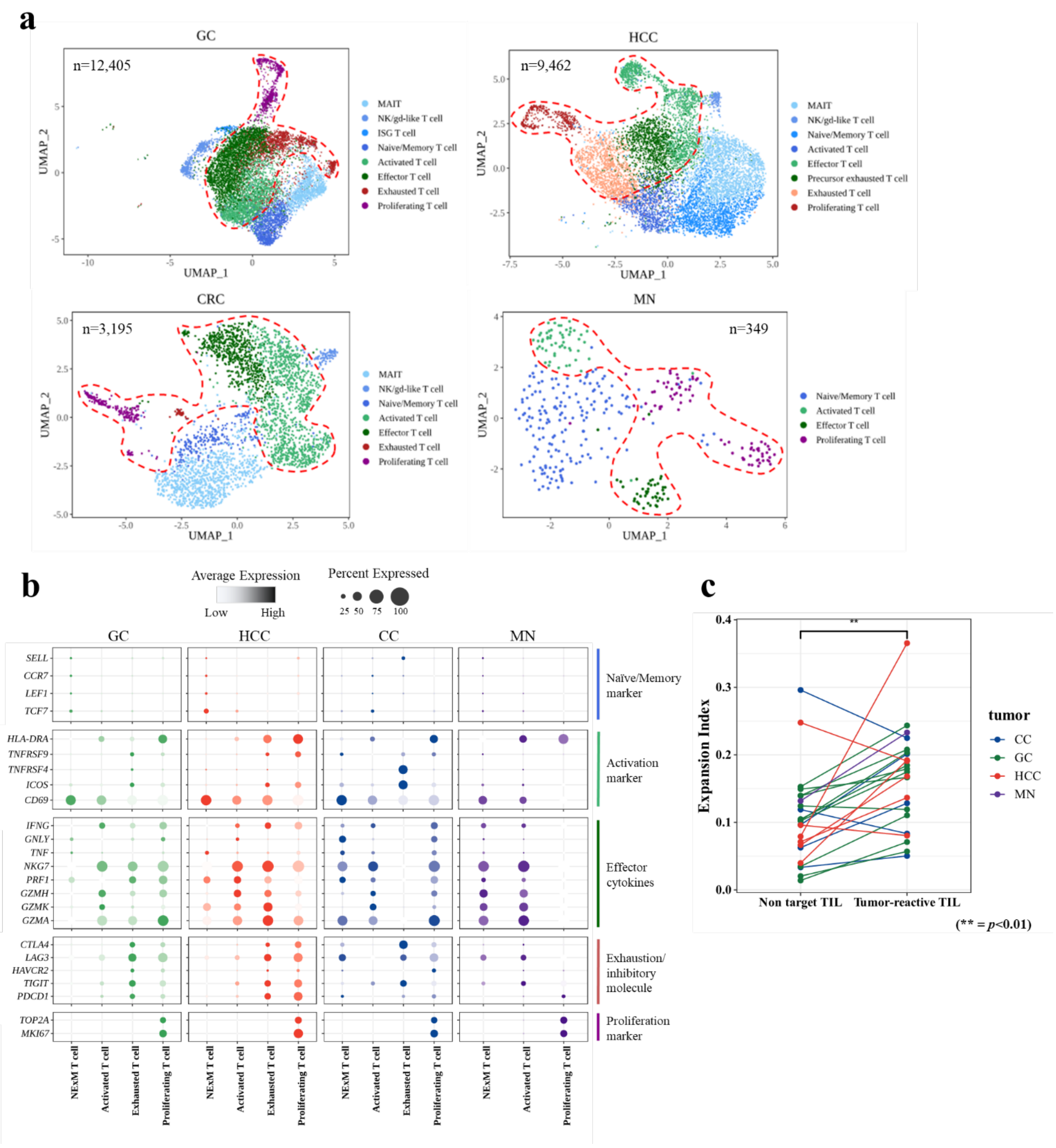

Supplementary Fig. 3. Single-cell analysis of CD8+ TILs from 25 cancer patients for the construction of a neoantigen selection model.

a Uniform Manifold Approximation and Projection (UMAP) plots of single-cell transcriptomic profiles of CD8+ TILs from 25 cancer patients (GC:11, HCC:6, CRC:2, and MN:2). Cluster-based T cell annotation was done based on the expression of marker genes shown in 2B. The red dashed lines represent putative tumor-reactive T cells including exhausted T cells, activated T cells, proliferative T cells, and effector T cells. A total of 25,411 CD8+ TILs were analyzed. **b** Marker genes used to annotate CD8+ TILs and their expression

heatmap. **c** Comparison of clonal expansion between tumor-reactive TILs and non-tumor reactive TILs (p <0.01).

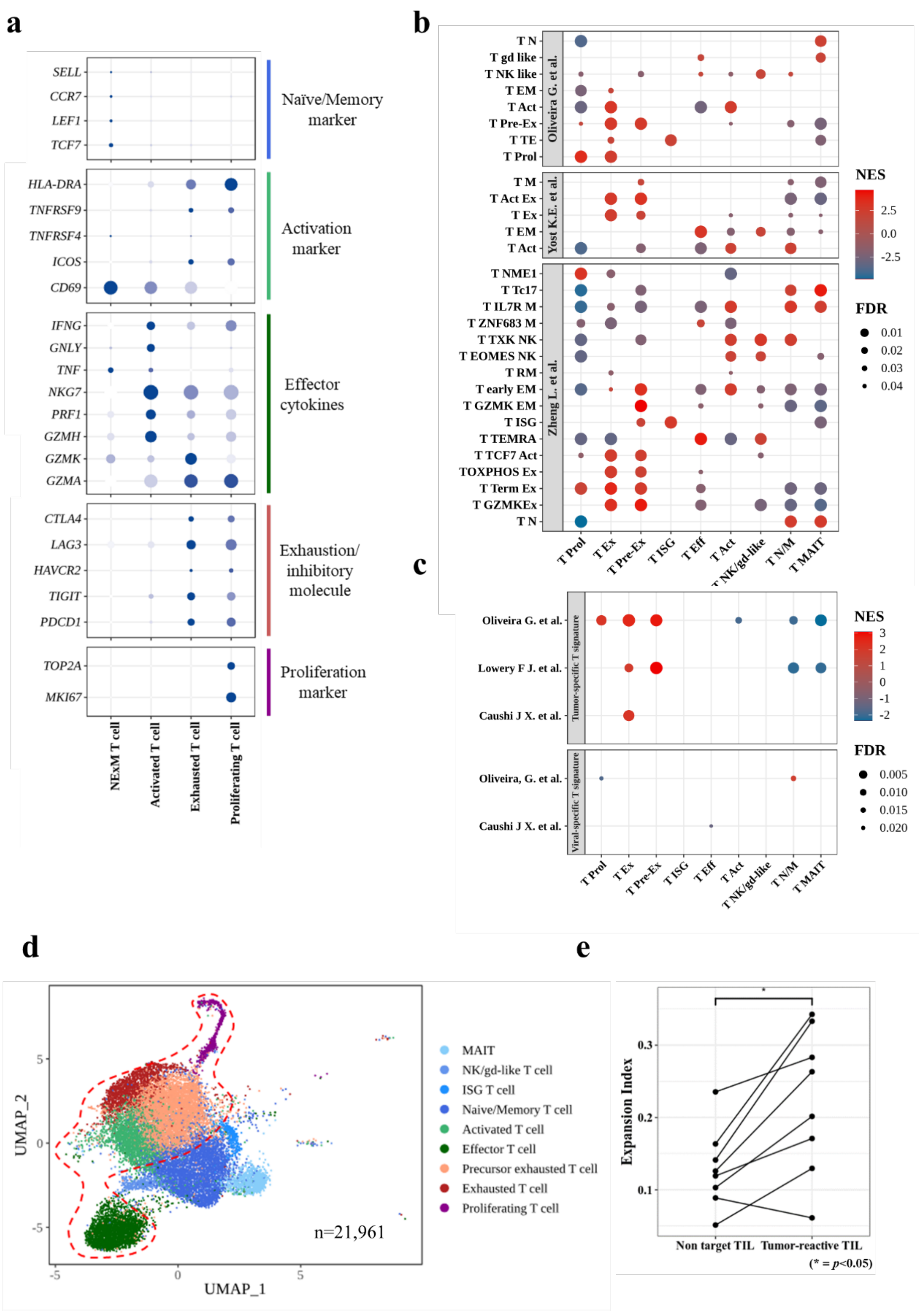

Supplementary Fig. 4. Single cell analysis of CD8+ TILs from 8 patients with HCC.

a Marker genes used to annotate CD8+ TILs and their expression heatmap. **b** Comparison of marker gene expression of CD8+ TILs subtypes between our group (x-axis) and 3 other groups (y-axis). **c** Expression of marker genes reported as tumor-reactive vs virus-reactive in T cell subtypes defined in this study. **d** Classification of CD8+ TILs from 8 patients with HCC undergoing platform validation. The red dashed lines

represent tumor-reactive T cells including exhausted T cells, activated T cells, proliferative T cells, and effector T cells. A total of 21,961 CD8+ TILs were analyzed. **e** Comparison of clonal expansion between tumor-reactive TILs and non-tumor reactive TILs. Clonal expansion detected in tumor-reactive TILs. *p* values are shown; statistical comparisons were performed using two-way ANOVA. * $p < 0.05$

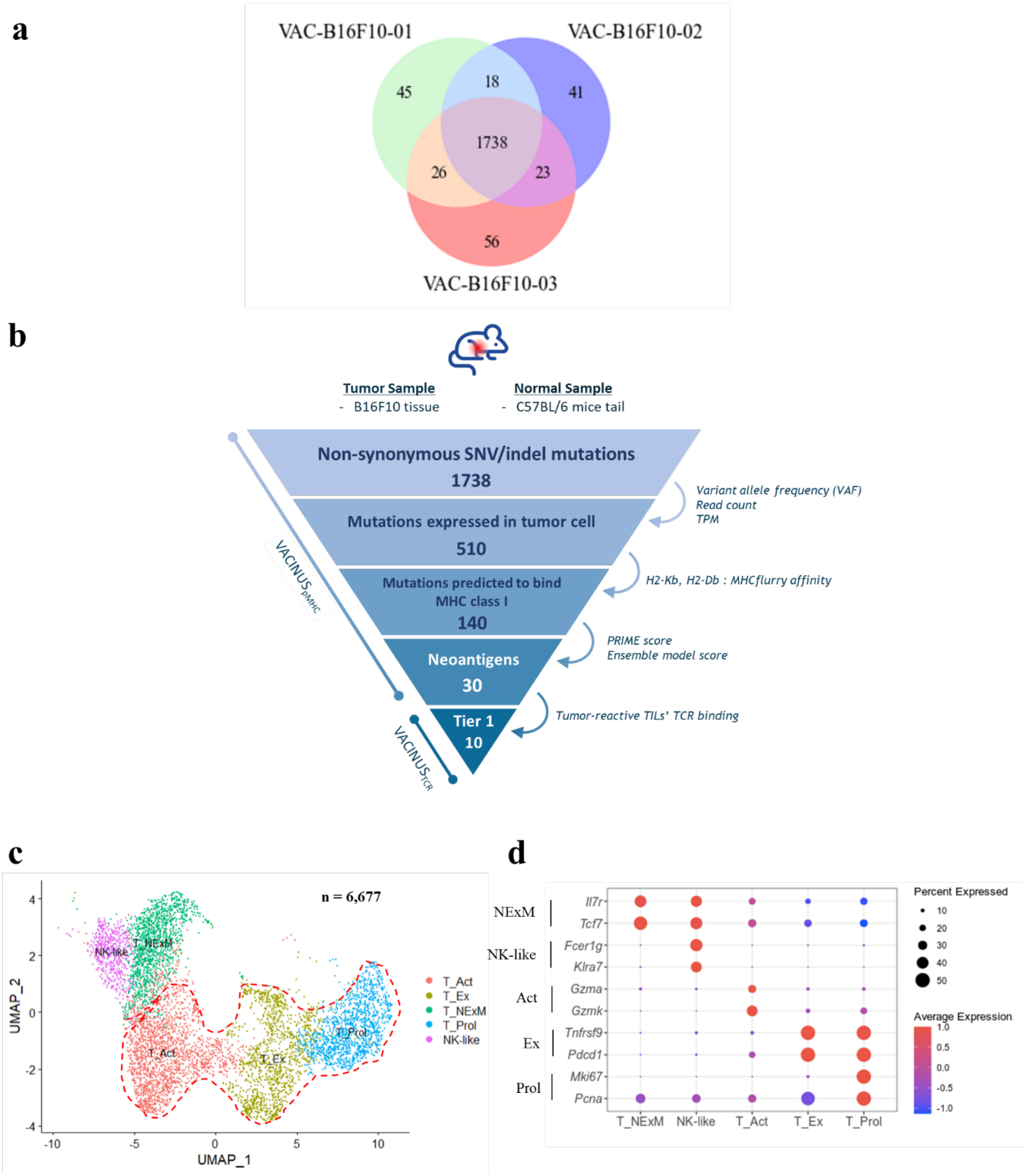

Supplementary Fig. 5. Neoantigens predicted from B16F10 tumor model and analysis of CD8+ TILs.

a Venn diagram of the mutations identified in tumor tissues from B16F10 tumor-bearing mice (n=3). A total of 1,738 nonsynonymous and InDel mutations were identified across the three replicates, accounting for 89.3% (1,738/1,947) of the total mutations. **b** Single-cell analysis of CD8+ TILs in B16F10 tumor tissue. A total of 6,677 T cells were analyzed, of which 4,701 cells (70.4%) were tumor-reactive TILs, including exhausted T cells, proliferative T cells, and activated T cells. **c** CD8+ TILs subpopulations based on marker genes and their expression heatmap. **d** Neoantigen selection was performed utilizing VACINUS$_{pMHC}$. Of a final selection of

30 neoantigens, 10 are predicted to be tier 1 neoantigens capable of interacting with tumor-reactive TILs.

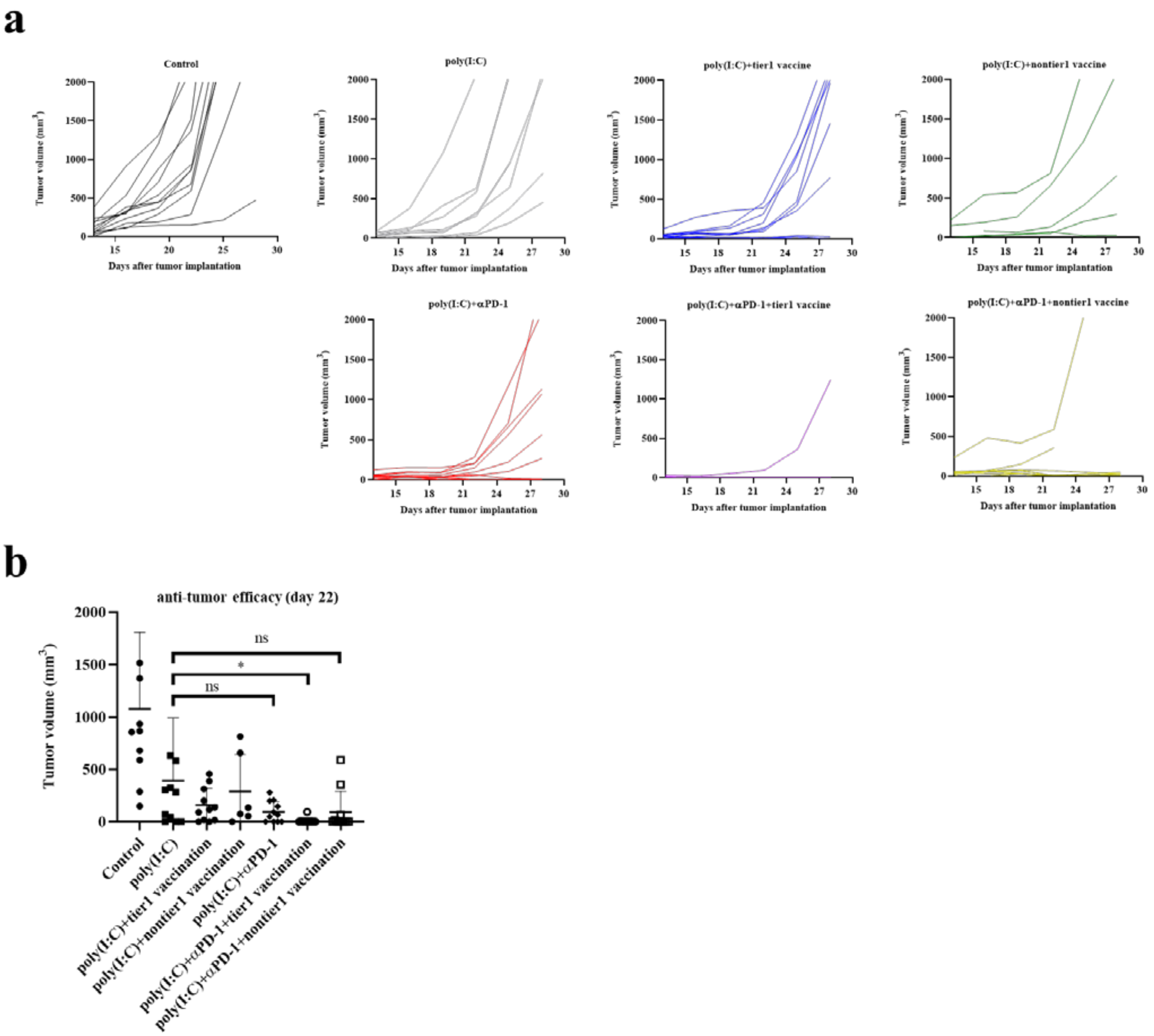

Supplementary Fig. 6. In vivo anti-tumor efficacy of candidate neoantigens.

Tumor growth curves of B16F10 melanoma bearing mice treated with the respective therapies (n = 11 mice per treatment group). **a** Tumor volume was measured at days 13, 16, 19, and 22. Tumor growth curves for individual mice in treatment groups until day 29. **b** Tumor volume was estimated at day 22, *p* values are shown; statistical comparisons were performed using two-way ANOVA. * p <0.05

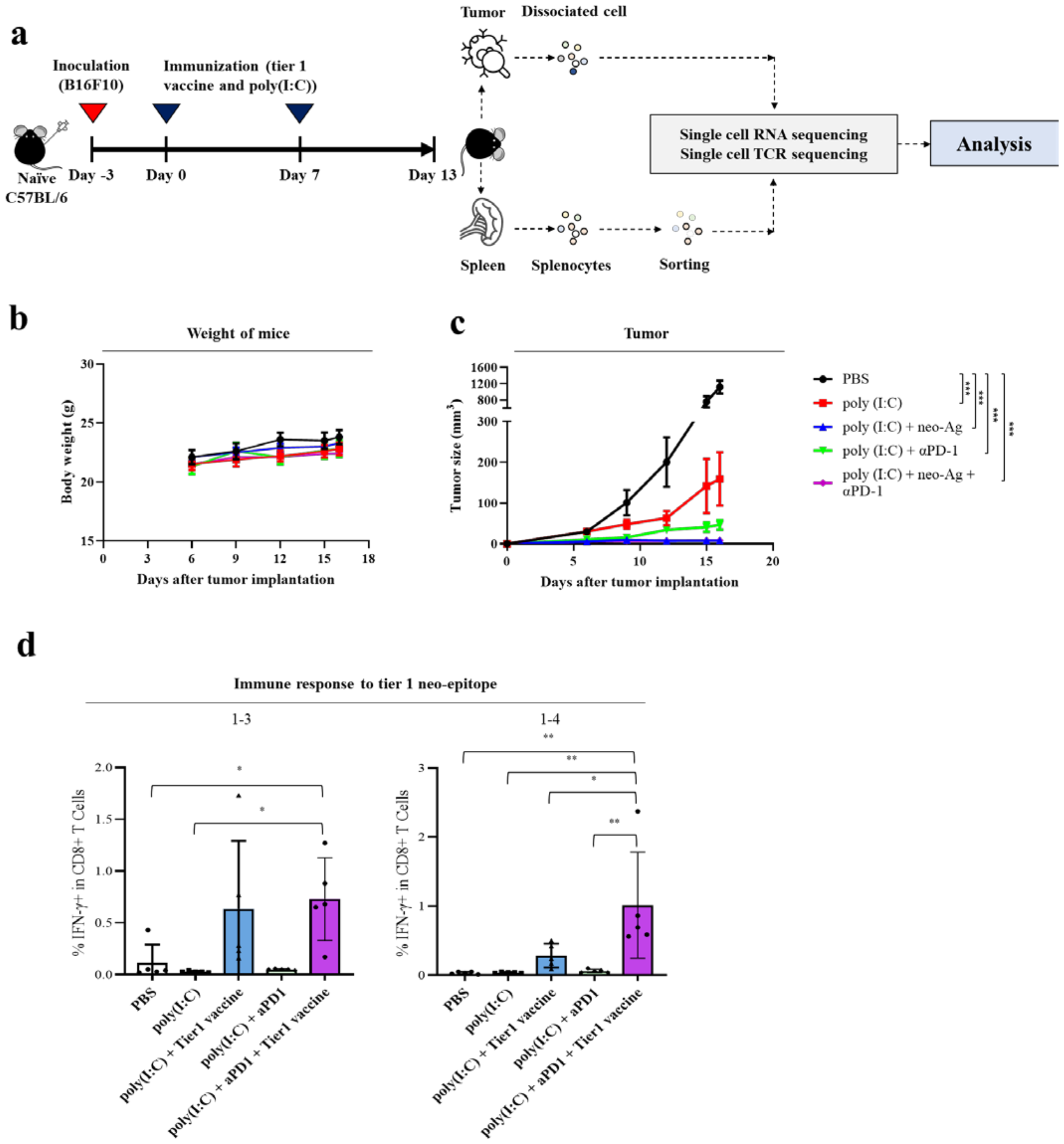

Supplementary Fig. 7. Antigen-specific immune responses after vaccination with tier 1 neoantigen.

a To understand the mechanism of anti-tumor responses following tier 1 vaccination, scRNA and TCR sequencing was performed on the spleen and tumor samples of B16F10 mouse model. 5 groups of mice were used (n= 5 mice per treatment group); control, poly(I:C), poly(I:C) +tier 1 vaccine, poly(I:C)+anti-PD-1, poly(I:C)+tier 1 vaccine+anti-PD-1 combination. At day 13, scRNA sequencing was performed on the tier 1 vaccine specific CD8+ T cells sorted from the spleen using dextramer staining and on whole tumor cells. The

vaccine was synthetic long peptides (SLP) consist of 27mer and contains 3 SLP of tier 1 epitopes. Vaccine was injected subcutaneously into right flank 2 times a week and anti-PD-1 was injected intraperitoneally with 5 times every 3 days after cancer cell inoculation. The concentration of peptide, poly(I:C) and anti-PD-1 was 100ug, 100ug, and 200ug per mouse, respectively. **b** The weight of B16F10 tumors bearing mice receiving the respective treatments (n = 5 mice per treatment group). **c** The survival of mice was measured at days 9, 12, 15, and 16. **d** The frequency of IFN-γ+CD8+ T cells in the spleen following restimulation with tier 1 neo-epitopes. Numbers indicate mean +/-SD of pooled data. p values are shown; statistical comparisons were performed using two-way ANOVA. *p<0.05, **p<0.01, ***p<0.001

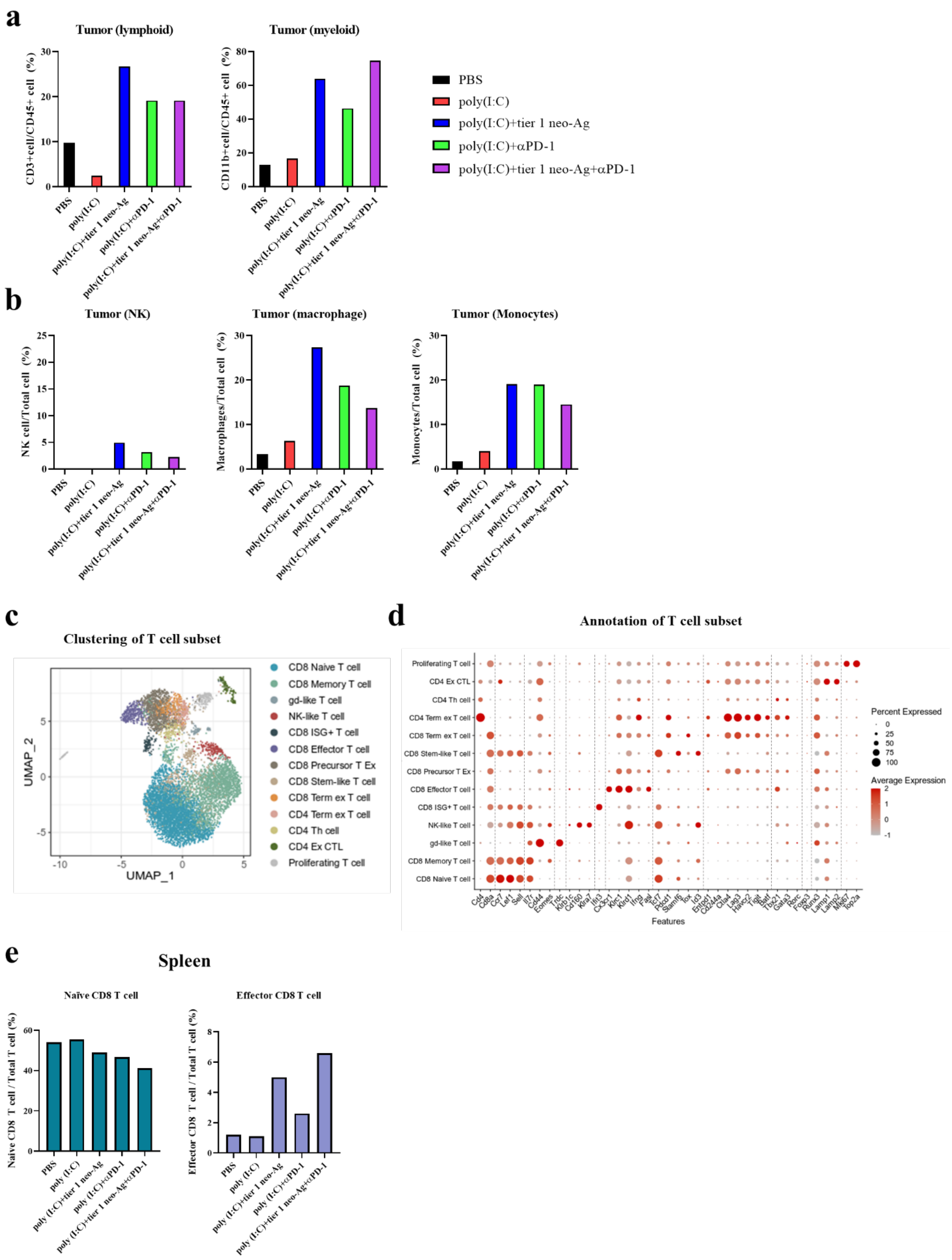

Supplementary Fig. 8. The profile of leukocytes and T cells after vaccination with tier 1 neoantigen.

a Percentage of CD3+ cells or CD11b+ cells in tumor leukocytes (CD45+ cells). **b** Percentage of NK cells, macrophages, monocytes in tumor. **c** Splenic and tumor T cell clusters defined by marker genes. **d** T cell

subsets defined by marker gene expression in splenocytes and tumor cells. **e** Ratio of naïve and effector CD8+ T cells in the spleen. ScRNA sequencing data were pooled 5 mice in all groups.

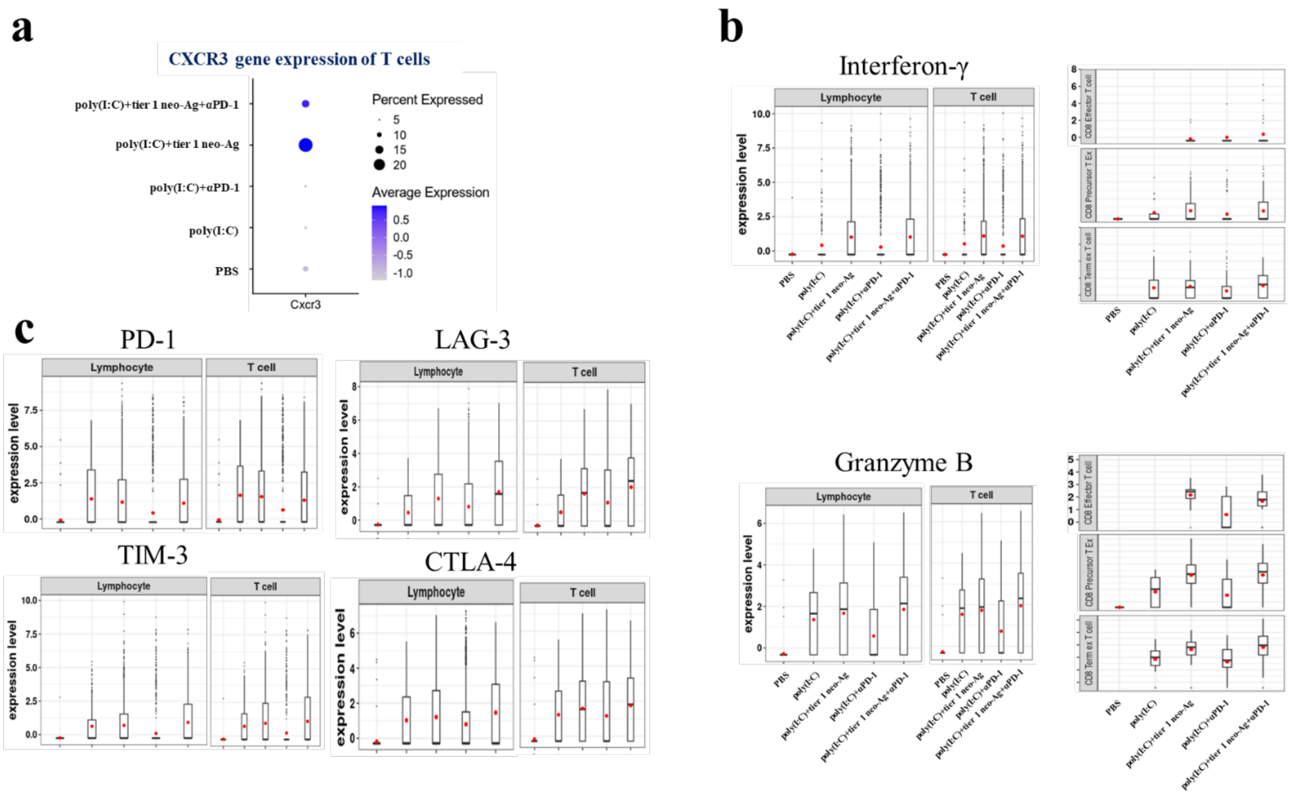

Supplementary Fig. 9. Analysis of functional T cells after vaccination with tier 1 neoantigen.

a CXCR3 expression in T cells. **b** IFN-γ and granzyme B expression levels in lymphocytes, T cell, effector CD8+ T cell, pre-exhausted CD8 T cell and terminally exhausted CD8+ T cell. **c** Expression levels of inhibitory molecules such as PD-1, LAG-3, TIM-3 and CTLA-4 in lymphocytes and T cell.

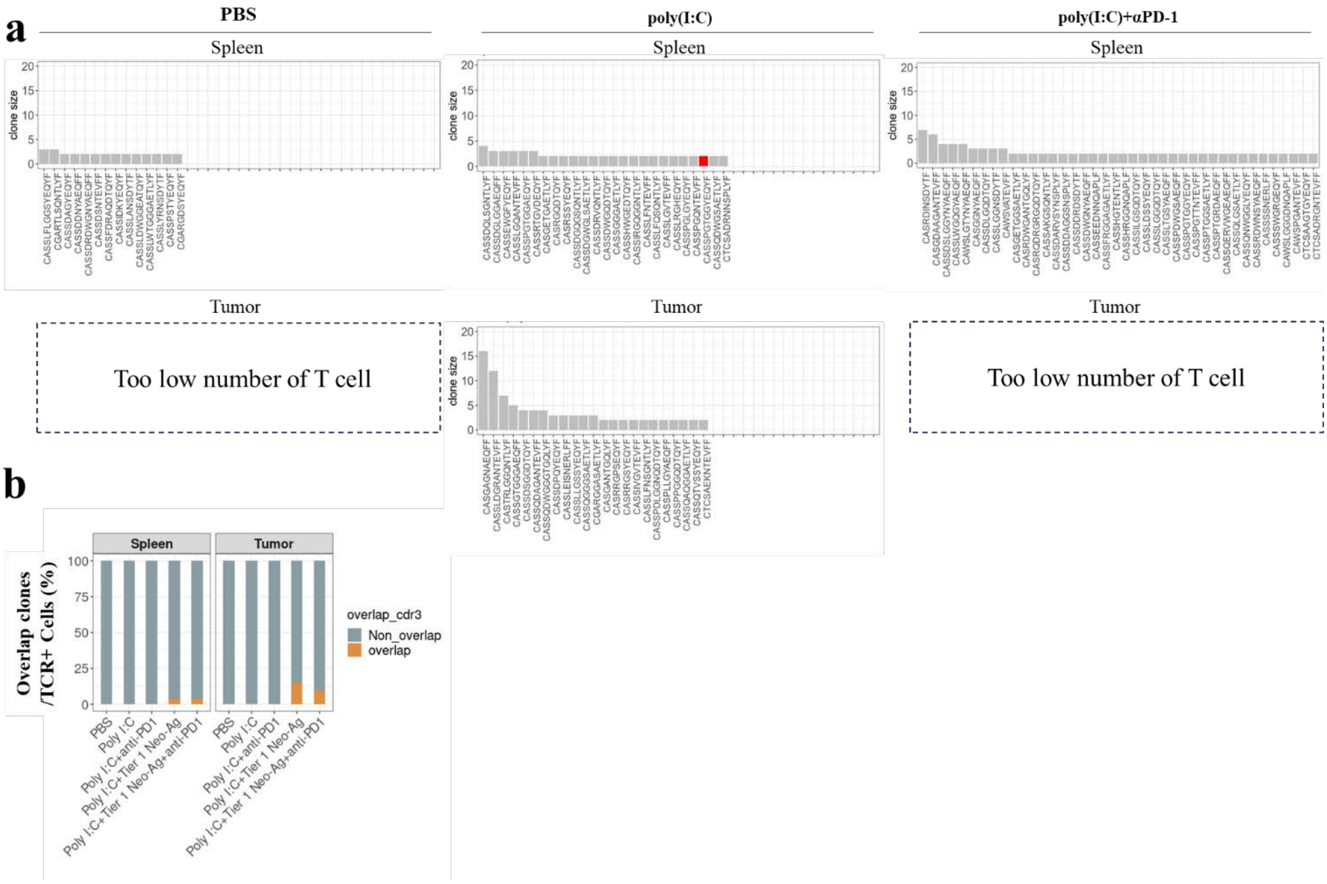

Supplementary Fig. 10. Overlapping CD8+ T cell clones in unvaccinated groups.

a Comparison of TCR CDR3 sequences between splenocytes and tumor cells in the un-vaccinated group. Sharing TCR sequences were shown in red and termed as overlapping clones. Splenocytes, CD8+dextramer+ antigen-specific T cell, were sorted from the spleen whereas dissociated whole tumor cells were sequenced. **b** Ratio of overlapping clones in the spleen and tumor following various treatments. Overlapping CD8+ T cell clones were found in the vaccinated group only.

Supplementary table 1. The bioinformatic toolsets used in VACINUS$_{pMHC}$ and VACINUS$_{TCR}$.

Tool name	Tool version	URL	Reference
BWA mem	v0.7.17-r1188	https://github.com/lh3/bwa	Heng Li , Richard Durbin, Fast and accurate short read alignment with Burrows–Wheeler transform, Bioinformatics, Volume 25, Issue 14, July 2009, Pages 1754–1760, https://doi.org/10.1093/bioinformatics/btp324
**STAR**	2.7.8a	https://github.com/alexdobin/STAR	Alexander Dobin and others, STAR: ultrafast universal RNA-seq aligner, Bioinformatics, Volume 29, Issue 1, January 2013, Pages 15–21, https://doi.org/10.1093/bioinformatics/bts635
**RSEM**	v1.3.1	https://github.com/deweylab/RSEM	Li B, Dewey CN. RSEM: accurate transcript quantification from RNA-Seq data with or without a reference genome. BMC Bioinformatics. 2011 Aug 4;12:323. doi: 10.1186/1471-2105-12-323. PMID: 21816040; PMCID: PMC3163565.
**OptiType**	v1.3.2	https://github.com/FRED-2/OptiType	Szolek, A, Schubert, B, Mohr, C, Sturm, M, Feldhahn, M, and Kohlbacher, O (2014). OptiType: precision HLA typing from next-generation sequencing data Bioinformatics, 30(23):3310-6.
**GATK**	gatk-v4.2.0.0	https://github.com/broadinstitute/gatk	McKenna A, Hanna M, Banks E, Sivachenko A, Cibulskis K, Kernytsky A, Garimella K, Altshuler D, Gabriel S, Daly M, DePristo MA. The Genome Analysis Toolkit: a MapReduce framework for analyzing next-generation DNA sequencing data. Genome Res. 2010 Sep;20(9):1297-303. doi: 10.1101/gr.107524.110. Epub 2010 Jul 19. PMID: 20644199; PMCID: PMC2928508.
**GATK mutect2**	gatk-v4.2.0.0	https://github.com/broadinstitute/gatk	Cibulskis, K., Lawrence, M., Carter, S. et al. Sensitive detection of somatic point mutations in impure and heterogeneous cancer samples. Nat Biotechnol 31, 213–219 (2013). https://doi.org/10.1038/nbt.2514
**Strelka2**	v2.9.2	https://github.com/Illumina/strelka	Kim, S., Scheffler, K., Halpern, A.L. et al. Strelka2: fast and accurate calling of germline and somatic variants. Nat Methods 15, 591–594 (2018). https://doi.org/10.1038/s41592-018-0051-x
**Seurat**	v2.5	https://github.com/tgen/seurat	Christoforides, A., Carpten, J.D., Weiss, G.J. et al. Identification of somatic mutations in cancer through Bayesian-based analysis of sequenced genome pairs. BMC Genomics 14, 302 (2013). https://doi.org/10.1186/1471-2164-14-302
**VEP**	v104.3	https://asia.ensembl.org/info/docs/tools/vep/index.html	McLaren, W., Gil, L., Hunt, S.E. et al. The Ensembl Variant Effect Predictor. Genome Biol 17, 122 (2016). https://doi.org/10.1186/s13059-016-0974-4
**MHCflurry**	v2.0.1	https://github.com/openvax/mhcflurry	O'Donnell et al., 2020, Cell Systems 11, 42–48 July 22, 2020 2020 The Author(s).Published by Elsevier Inc. https://doi.org/10.1016/j.cels.2020.06.010
**Prime**	v1.0	https://github.com/GfellerLab/PRIME	Schmidt et al., Prediction of neo-epitope immunogenicity reveals TCR recognition determinants and provides insight into immunoediting. Cell Reports Medicine, 2021.
**Antigen garnish**	v2.3.0	https://github.com/andrewrech/antigen.garnish	Richman LP, Vonderheide RH, and Rech AJ. Neoantigen dissimilarity to the self-proteome predicts immunogenicity and response to immune checkpoint blockade. Cell Systems. 2019.
**TCRpMHC models**	v1.0	https://services.healthtech.dtu.dk/services/TCRpMHCmodels-1.0/	Jensen, K.K., Rantos, V., Jappe, E.C. *et al.* TCRpMHCmodels: Structural modelling of TCR-pMHC class I complexes. *Sci Rep* **9**, 14530 (2019). https://doi.org/10.1038/s41598-019-50932-4
**pMTnet**	v1.0	https://github.com/tianshilu/pMTnet	Lu, T., Zhang, Z., Zhu, J. et al. Deep learning-based prediction of the T cell receptor–antigen binding specificity. Nat Mach Intell 3, 864–875 (2021). https://doi.org/10.1038/s42256-021-00383-2
**FoldX**	v5.0	https://foldxsuite.crg.eu/	Delgado J, Radusky LG, Cianferoni D, et al. FoldX 5.0: working with RNA, small molecules and a new graphical interface. Bioinformatics 2019;35:4168–9.

Supplementary table 2. Twenty features considered for the ensemble model.

Feature	Selection	Description
MHCflurry processing score	O	Likelihood of a peptide being detected in mass spectrometry. Higher scores indicate more favorable processing by proteasome for detection.
MHCflurry affinity	O	Predicted score for peptide-HLA binding affinity
MHCflurry affinity percentile	O	Percentile of predicted scores for peptide-HLA binding affinity
MHCflurry presentation score	O	A score for the likelihood of presentation of the peptide. This score is a simple logistic regression model that combines the binding affinity prediction with the processing score to give a composite prediction.
MHCflurry presentation percentile	O	Percentile of scores for the likelihood of presentation of peptide.
TPM sum	O	The sum of transcripts per million, a quantitative measure of gene expression.
WTS read count	O	Number of reads that passed quality control from WTS
WES VAF	O	Variant allele frequency from whole exome sequencing.
WTS VAF	O	Variant allele frequency from whole transcriptome sequencing.
Foreignness score	O	Neoantigen foreignness threshold. Value of 0 to 1 indicating the TCR recognition probability, calculated by summing alignments in IEDB immunogenic peptides, with 1 indicating greater homology to immunogenic peptides.
Dissimilarity	O	Value of 0 to 1 indicating alignment to the self-proteome, calculated in an analogous manner to neoantigen foreignness, with 1 indicating greater dissimilarity.
Structure-based energy score	O	FoldX-based binding energy score of a peptide-MHC pair based on structural modeling
SASA value	O	Solvent accessible surface area (SASA) is calculated by using FreeSASA software.
WES VAF/tumor purity	X	Allele frequency in WES divided by tumor purity.
WTS VAF/tumor purity	X	Allele frequency in WTS divided by tumor purity.
Mutation position	X	Where the mutation occurred in the peptide.
Agretopicity	X	The ratio of MHC binding affinity between mutant and normal peptide.
PRIME score	X	The PRIME score predicts the immunogenicity of class I epitopes, combining HLA-I binding and T-cell receptor recognition likelihood.
Peptide length	X	Peptide length
Structure-based binding confidence	X	Structure modeling prediction confidence score.

Supplementary table 3. The validation of VACINUS$_{pMHC}$ workflow using immunogenic *in vitro* screening from PBMCs of HCC patients.

Pt ID	Screened Neoantigens (n)	Immunogenic Neoantigens (n)	Neoantigen Positivity (%)
**HCC 1**	14	2	14.3%
**HCC 2**	16	2	12.5%
**HCC 3**	18	7	38.9%
**HCC 4**	17	7	41.2%
**HCC 5**	12	3	25.0%
**HCC 6**	5	0	0.0%
**HCC 7**	18	4	22.2%
**HCC 8**	18	5	27.8%
**Total**	118	30	25.4%

Supplementary table 4. DEG analysis of tumor-reactive TILs in 8 patients with HCC.

Supplementary table 5. Validation of predicted neoantigens in 8 patients with HCC.

Pt ID	Predicted Neoantigens (n)		Validated Neoantigens (n)		tier 1 Positivity	nontier 1 Positivity	Neoantigen Positivity
	tier 1	nontier 1	tier 1	nontier 1			
**HCC 11**	8	6	0	2	0.0%	33.3%	14.3%
**HCC 13**	6	10	2	0	33.3%	0.0%	12.5%
**HCC 15**	3	15	2	5	66.7%	33.3%	38.9%
**HCC 16**	10	7	4	3	40.0%	42.9%	41.2%
**HCC 17**	7	5	2	1	28.6%	20.0%	25.0%
**HCC 19**	2	3	0	0	0.0%	0.0%	0.0%
**HCC 20**	6	12	1	3	16.7%	25.0%	22.2%
**HCC 21**	6	12	2	3	33.3%	25.0%	27.8%
**Total**	48	70	13	17	27.1%	24.3%	25.4%

Supplementary table 6. Thirty neoantigens identified in mouse B16F10 tumor model using VACINUS$_{pMHC}$ and 10 tier 1 neoantigens.

Neoantigen ID	Chromosome	Consequence	Gene	Neoantigen	Synthetic Long Peptide Sequence (MT)	Synthetic Long Peptide Sequence (WT)	Ensemble score	Tier	Tumor-reactive TILs CDR3B	Matched TILs
B16-1_1	chr9_67913975_T/C	missense variant	Vps13c	SLPTNAVVV, SSLPTNAVVV	LKAPVIVIPQSSL**P**TNAVVVDLGLIRV	LKAPVIVIPQSSL**S**TNAVVVDLGLIRV	0.3874	1	CASSFHKNYAEQFF, CASSRDRGDEQYF	T Ex, T Act
B16-1_2	chrX_71441088_G/T	missense variant	Cd99l2	RAQSNPMEL	ATTTTRRPGTTRA**Q**SNPMELDGFDLED	ATTTTRRPGTTRA**P**SNPMELDGFDLED	0.2077	1	CASSSGHNQAPLF, CASSMGEGEQYF	T Act, T Prol
B16-1_3	chr1_7163330_C/T	missense variant	Pcmtd1	VSFAPLVQL	KNILAVSFAPLVQ**L**SKNDNGTPDSVGL	KNILAVSFAPLVQ**P**SKNDNGTPDSVGL	0.1928	1	CASSFHKNYAEQFF, CASSDAGRDSDYTF	T Ex, T Act
B16-1_4	chr8_105231172_C/T	missense variant	D230025D16Rik	CIIRNVQVL	PLAQAVAILQKHC**C**IIRNVQVLYSEQS	PLAQAVAILQKHC**R**IIRNVQVLYSEQS	0.1648	1	CASSLDPANTEVFF	T Ex
B16-1_5	chr14_122478675_A/T	missense variant	Zic2	KSYTLPSSL	KPYLCKMCDKSYT**L**PSSLRKHMKVHES	KPYLCKMCDKSYT**H**PSSLRKHMKVHES	0.1581	1	CASSLGGRPEVFF, CASSFHKNYAEQFF, CASSILGSQNTLYF, CASSRLGGNQDTQYF	T Act, T Ex, T Prol, T Ex
B16-1_6	chr16_36919742_A/C	missense variant	Golgb1	SSPADVQSL	QRAAAPSAASSPA**D**VQSLKKAMSSLQN	QRAAAPSAASSPA**E**VQSLKKAMSSLQN	0.1552	1	CASSPLPGPYEQYF, CASSLGGRPEVFF, CASSPEAGYNERLFF, CASSLDPANTEVFF, CASSLGTGYAEQFF, CASSILGSQNTLYF, CASSLGGDSQYF	T Prol, T Act, T Ex, T Ex, T Ex, T Prol, T Act
B16-1_7	chr9_31411333_A/C	missense variant	Nfrkb	SAPRASTTA	SKTAMSSPGNSAP**R**ASTTAVIQNVTGQ	SKTAMSSPGNSAP**S**ASTTAVIQNVTGQ	0.1461	1	CASSFHKNYAEQFF, CASSDAGRDSDYTF	T Ex, T Act
B16-1_8	chr19_10619230_C/A	missense variant	Ddb1	VLMINGEEV	LVLSFVGQTRVLM**I**NGEEVEETELMGF	LVLSFVGQTRVLM**L**NGEEVEETELMGF	0.1446	1	CASSFHKNYAEQFF	T Ex
B16-1_9	chr1_5098048_A/C	missense variant	Atp6v1h	MAARIIATL	DPFTVHMAARIIA**T**LAAWGKELMEGSD	DPFTVHMAARIIA**K**LAAWGKELMEGSD	0.1423	1	CASSMISGNTLYF, CASSLGGRPEVFF, CASSFHKNYAEQFF, CASSLDPANTEVFF, CASSLGGDSQYF	T Ex, T Act, T Ex, T Ex, T Act
B16-1_10	chr8_45023807_A/G	missense variant	Fat1	IAMQNTTQL	EKFSMDHKTGTIA**M**QNTTQLRSRYELT	EKFSMDHKTGTIA**I**QNTTQLRSRYELT	0.1417	1	CASSDAGGGNQDTQYF, CASSFHKNYAEQFF, CASSDAGEQDTQYF	T Prol, T Ex, T Act
B16-3_1	chr7_45127513_G/C	missense variant	Rpl13a	AAIVGKQVL, AAIVGKQVLL	GRGHLLGRLAAIV**G**KQVLLGRKVVVVR	GRGHLLGRLAAIV**A**KQVLLGRKVVVVR	0.6796			
B16-3_2	chr1_59483870_G/C	missense variant	Fzd7	VAHVAAFL, VAVAHVAAF	SGCYFMVAVAHVA**A**FLLEDRAVCVERF	SGCYFMVAVAHVA**G**FLLEDRAVCVERF	0.5666			
B16-3_3	chr2_144252491_C/T	missense variant	Snx5	AAFQKNLIEM	ELINFKRKRVAAF**Q**KNLIEMSELEIKH	ELINFKRKRVAAF**R**KNLIEMSELEIKH	0.3702			
B16-3_4	chr4_155991934_C/A	missense variant	B3galt6	VHYLRLSL	VLSADLVHYLRLS**L**EYLRAWHSEDVSL	VLSADLVHYLRLS**R**EYLRAWHSEDVSL	0.3261			
B16-3_5	chr14_50951495_T/G	missense variant	Pnp	VVMEYENL	VFGFSLITNKVVM**E**YENLEKANHMEVL	VFGFSLITNKVVM**D**YENLEKANHMEVL	0.2835			
B16-3_6	chr7_142376597_C/T	missense variant	Ctsd	VSFANAVV	SYYTVFDRDNNRV**S**FANAVVL	SYYTVFDRDNNRV**G**FANAVVL	0.2708			
B16-3_7	chr8_111297606_C/A	missense variant	Rfwd3	VIYSHLQV	SEMDHEVIYSHLQ**V**PLEGTIEPATPTE	SEMDHEVIYSHLQ**G**PLEGTIEPATPTE	0.2165			
B16-3_8	chr4_155886970_G/A	missense variant	Ints11	RTFANNPGPM	EFKHIKAFDRTFA**N**NPGPMVVFATPGM	EFKHIKAFDRTFA**D**NPGPMVVFATPGM	0.2162			
B16-3_9	chr10_76285628_T/C	missense variant	Dip2a	LVFVVGRL	GFIGPDNLVFVVG**R**LDGLMVVGVRRHN	GFIGPDNLVFVVG**K**LDGLMVVGVRRHN	0.2115			
B16-3_10	chr7_44552134_T/C	missense variant	Nr1h2	SGFRYNVL	ELCRVCGDKASGF**R**YNVLSCEGCKGFF	ELCRVCGDKASGF**H**YNVLSCEGCKGFF	0.1993			
B16-3_11	chr6_39364594_G/A	missense variant	Slc37a3	FSYSLLHV	VFLLTFFSYSLLH**V**SRKTFSNVKVSIS	VFLLTFFSYSLLH**A**SRKTFSNVKVSIS	0.1949			
B16-3_12	chr19_40607346_A/C	missense variant	Tctn3	VSVRQTNL	TNGTFGIQKVSVS**V**RQTNLTVKPGVSL	TNGTFGIQKVSVS**F**RQTNLTVKPGVSL	0.1923			
B16-3_13	chr13_69615465_A/T	missense variant	Nsun2	KILRMSPL	LAWHTNLSRKILR**M**SPLLAKFHQFLVS	LAWHTNLSRKILR**K**SPLLAKFHQFLVS	0.188			
B16-3_14	chr1_58476516_A/C	missense variant	Orc2	VVPSFSAEI	RVDQKTLHNLLRK**V**VPSFSAEIERLNQ	RVDQKTLHNLLRK**F**VPSFSAEIERLNQ	0.1868			
B16-3_15	chr1_93316801_C/T	missense variant	Pask	ANFIFRQL	FIDHHPCLDEPLA**N**FIFRQLVSAVGYL	FIDHHPCLDEPLA**S**FIFRQLVSAVGYL	0.1842			
B16-3_16	chr17_50963696_C/A	missense variant	Tbc1d5	VKITFPEM	DKELRSMIEQDVK**I**TFPEMQFFQQENV	DKELRSMIEQDVK**R**TFPEMQFFQQENV	0.1781			
B16-3_17	chr14_65813948_T/A	missense variant	Pbk	AAVILRDAL	DSGSPFPAAVILR**D**ALHMARGLKYLHQ	DSGSPFPAAVILR**V**ALHMARGLKYLHQ	0.1712			
B16-3_18	chr10_57496168_A/T	missense variant	Hsf2	NMYGFRNV	ASFVRQLNMYGFR**N**VVHIESGIIKQER	ASFVRQLNMYGFR**K**VVHIESGIIKQER	0.1652			
B16-3_19	chr2_104434629_G/A	missense variant	Hipk3	ATLTFEGM	QVTPMAPAAATLT**F**EGMAGSQRLGDWG	QVTPMAPAAATLT**S**EGMAGSQRLGDWG	0.1646			
B16-3_20	chr3_90515942_C/G	missense variant	S100a13	VVCTFFTF	TLTELEAAIETVV**C**TFFTFAGREGRKG	TLTELEAAIETVV**S**TFFTFAGREGRKG	0.1616			

Supplementary table 7. DEG analysis of TILs in B16F10 tumor.

Supplementary table 8. Neoepitopes with positive immunogenicity.

Name	AA seq	Gene	Neoantigen	Public report
B16-1-3-E1	VSFAPLVQL	Pcmtd1	tier 1	X
B16-1-4-E1	CIIRNVQVL	D230025D16Rik	tier 1	X
B16-1-7-E1	SAPRASTTA	Nfrkb	tier 1	X
B16-3-1-E1	AAIVGKQVL	Rpl13a	nontier 1	Pool only (Caudana P et al. 2019)
B16-3-5-E1	VVMEYENL	Pnp	nontier 1	prediction only (Dhuey E et al. 2022)
B16-3-6-E1	VSFANAVV	Ctsd	nontier 1	X
B16-3-15-E1	ANFIFRQL	Pask	nontier 1	X
B16-3-16-E1	VKITFPEM	Tbc1d5	nontier 1	X
B16-3-18-E1	NMYGFRNV	Hsf2	nontier 1	prediction only (Dhuey E et al. 2023)

Supplementary table 9. Marker genes for specific immune cell type.

Cell type			Phenotype	Gene
Immune			CD45	*Ptprc*
Myeloid			CD11b	*Itgam*
Myeloid	granulocyte	Neutrophil	MRP8, MRP14	*S100a8, S100a9*
		mast cell	FCER1a+, CD117+,	*Fcer1a, Kit*
		Basophil	FCER1a-, CD117+, CD49b+	*Fcer1a, kit, Cd49b*
	Dendritic cell	cDC1	FLT3+, IRF8^{hi}, XCR1+	*Flt3, Irf8, Xcr1*
		cDC2	FLT3+, IRF8^{lo}, CD209+	*Flt3, Irf8, Cd209*
	Macrophage	Macrophage	F4/80+	*Adgre1*
	Monocytes	Monocytes	CD14+, F4/80-	*Cd14, Adgre1-*
Lymphoid	NK cell	NK cell	NK1.1+, NKG2D+	*Klrb1c, Klrk1*
	B cell	B cell	CD79a+, CD79b+	*Cd79a, Cd79b*
	T cell	CD3+ T cell	CD3+	*Cd3*
		CD4+ T cell	CD4+	*Cd4*
		CD8+ T cell	CD8+	*Cd8*

Supplementary table 10. DEG analysis of T cell subsets in B16F10 bearing mice after tier 1 vaccination.
